# Supplementary material for: Comprehensive analysis of the human ESCRT-III-MIT domain interactome reveals new cofactors for cytokinetic abscission
Source: eLife. 2022 Sep 15;11:e77779. doi: 10.7554/eLife.77779 (PMC9477494; doi:10.7554/eLife.77779)
Supplement: Figure 2—figure supplement 6—source data 1. [file elife-77779-fig2-figsupp6-data1.zip › Figure 2-figure supplement 6-source data 1/Figure 2-figure supplement 6_uncroppedblots.pdf]

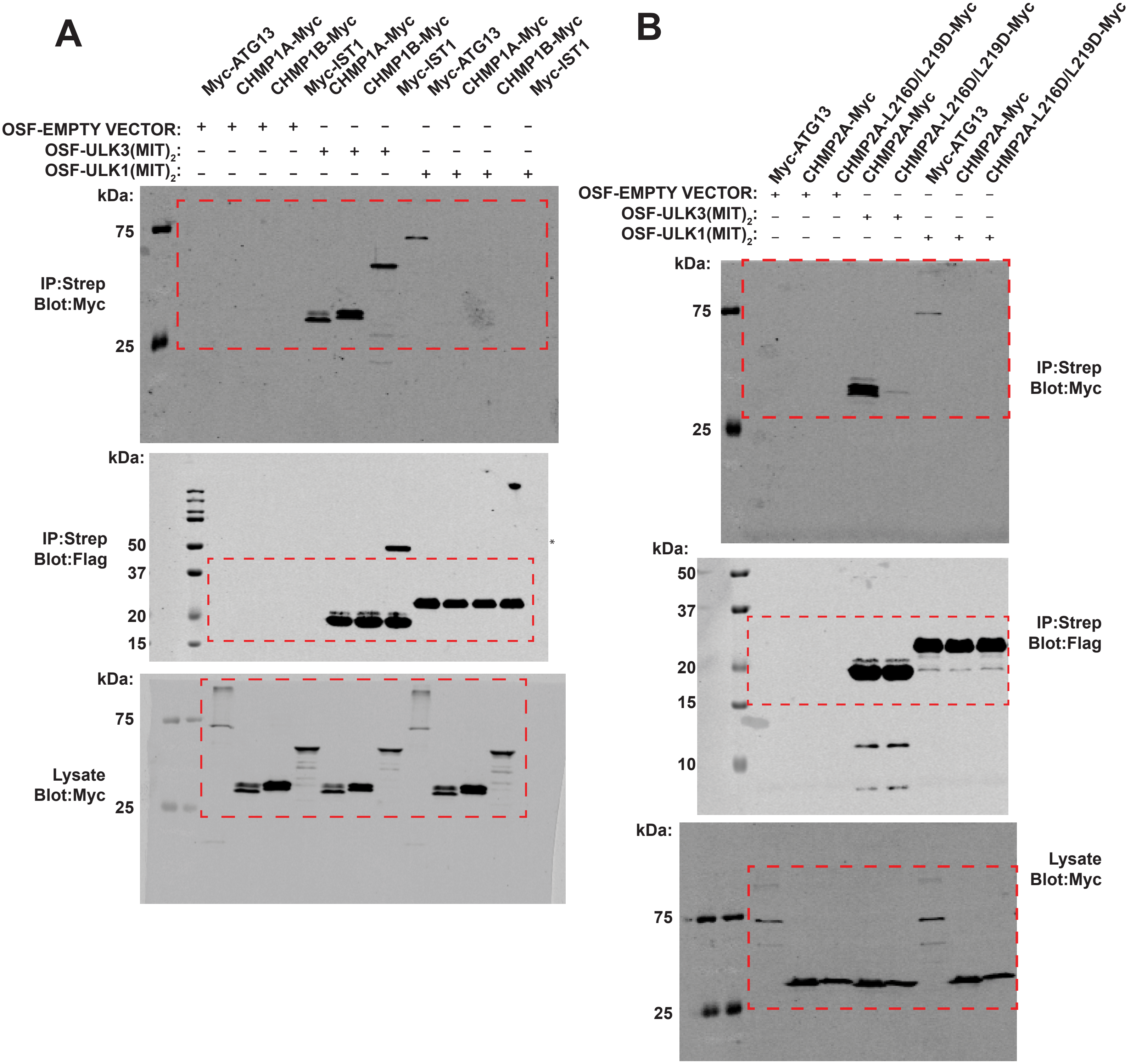

Uncropped Western blots for Figure 2-figure supplement 6. (**A**) Uncropped blots for Figure 2-figure supplement 6A. Asterisk in middle panel denotes cross-reactivity with the band corresponding to IST1 and/or its secondary antibody. (**B**) Uncropped blots for Figure 2-figure supplement 6B.
