# Supplementary figures and images for: Comprehensive analysis of the human ESCRT-III-MIT domain interactome reveals new cofactors for cytokinetic abscission
Source: eLife. 2022 Sep 15;11:e77779. doi: 10.7554/eLife.77779 (PMC9477494; doi:10.7554/eLife.77779)

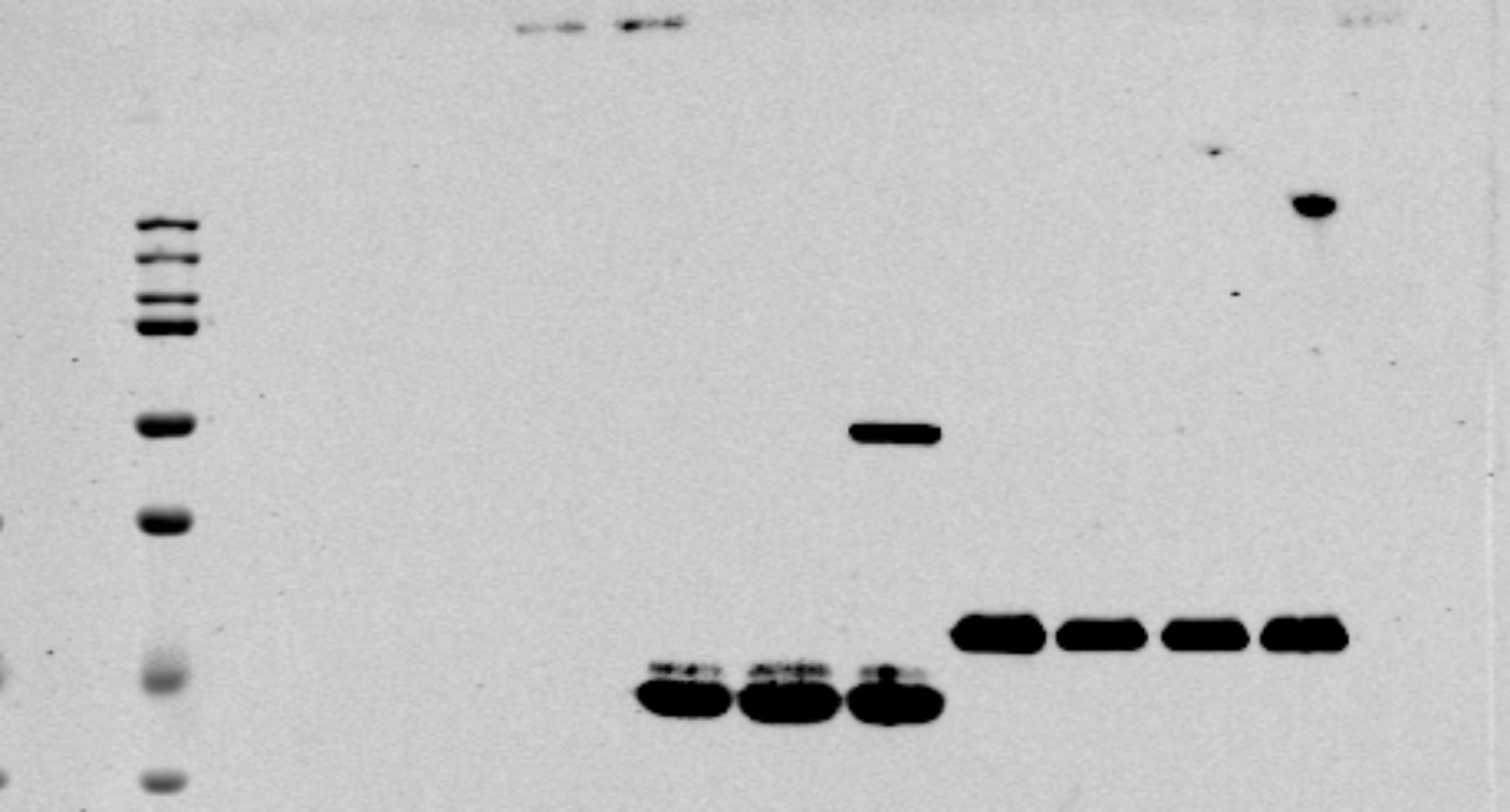

Supplement: Figure 2—figure supplement 6—source data 1. [file elife-77779-fig2-figsupp6-data1.zip › Figure 2-figure supplement 6-source data 1/Figure 2-figure supplement 6A_rawimages/Flag_figure2_figuresupplement 6A.tif]

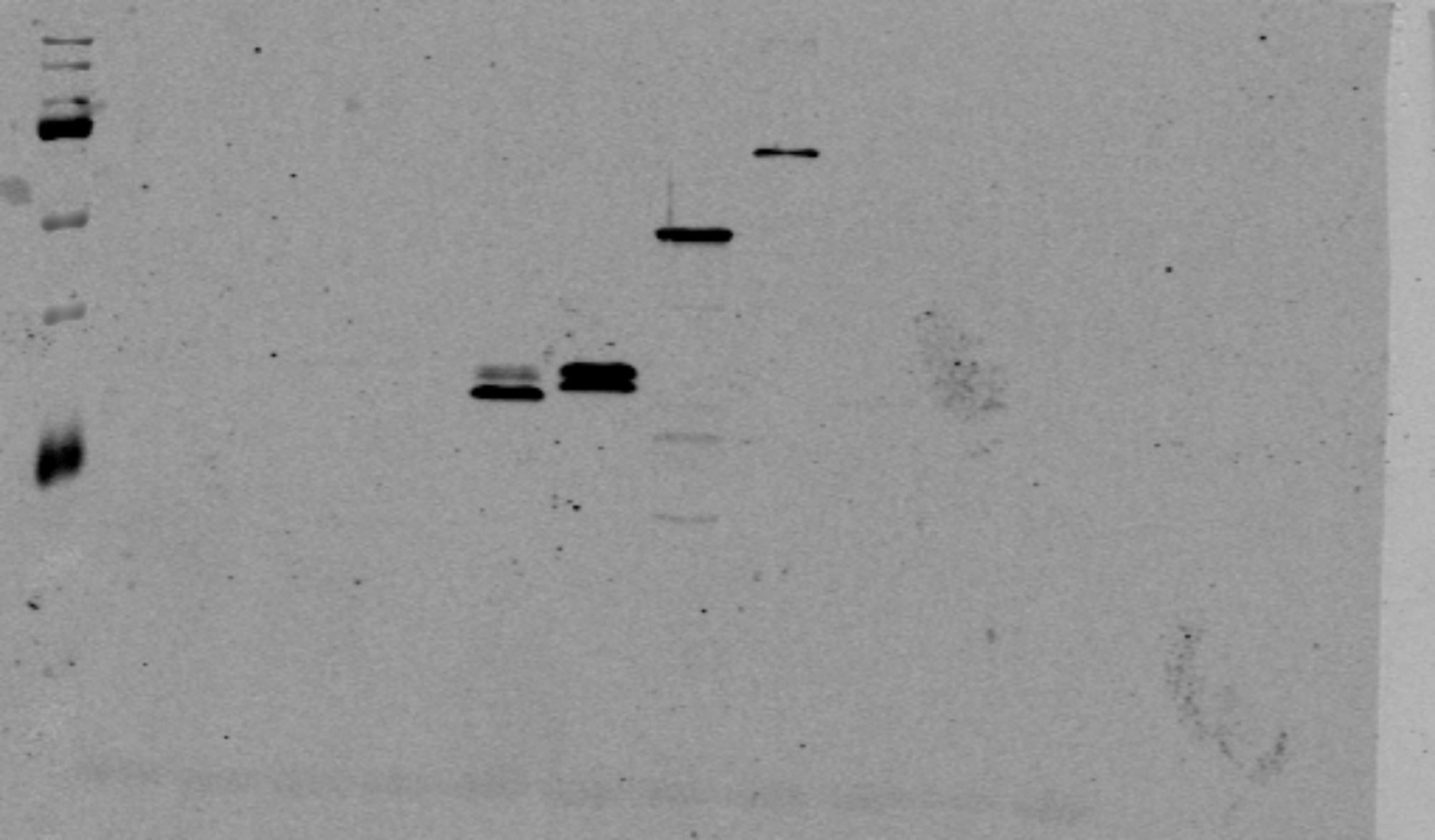

Supplement: Figure 2—figure supplement 6—source data 1. [file elife-77779-fig2-figsupp6-data1.zip › Figure 2-figure supplement 6-source data 1/Figure 2-figure supplement 6A_rawimages/IPstrepBlotMyc_figure2_figuresupplement 6A.tif]

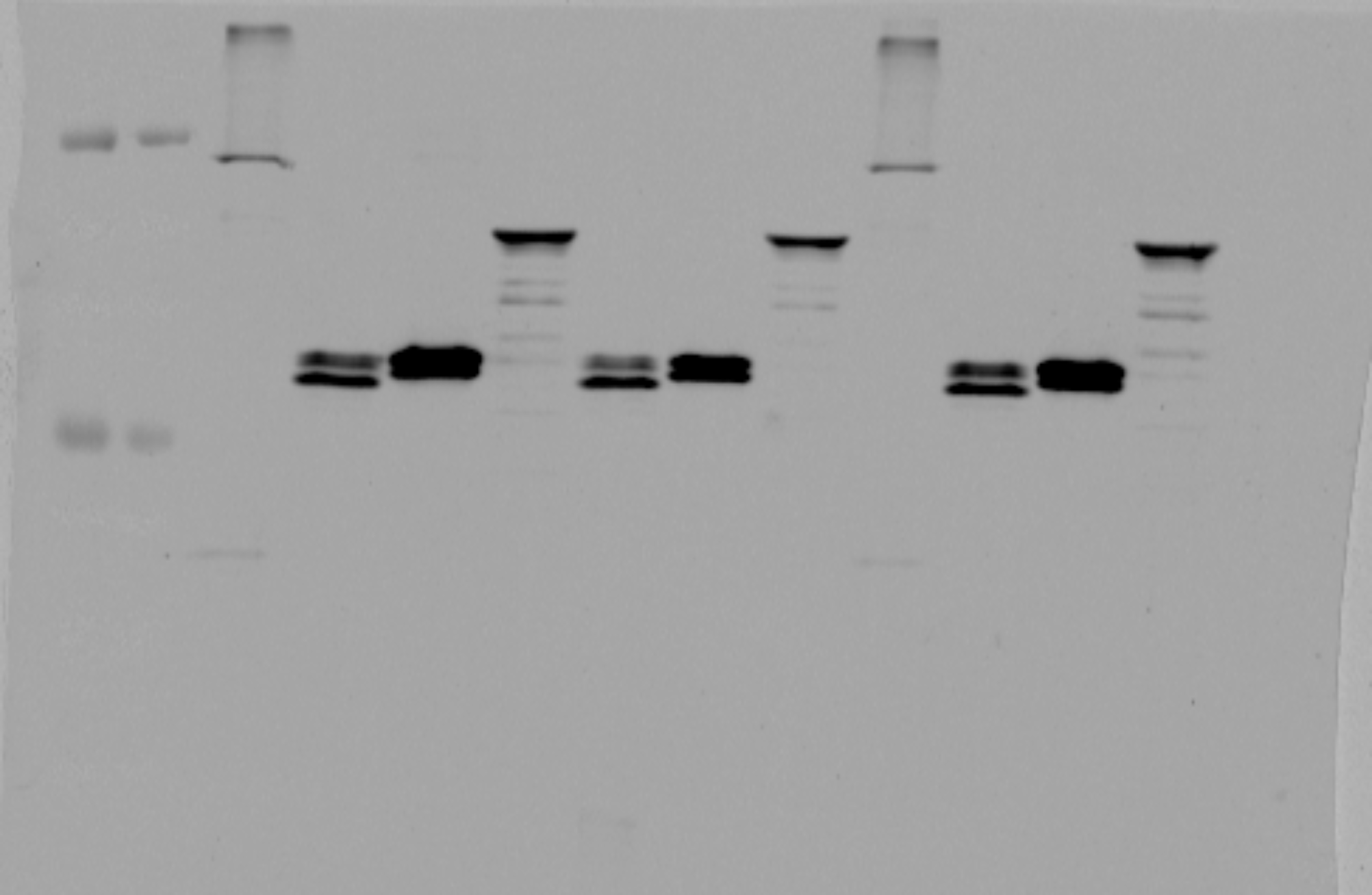

Supplement: Figure 2—figure supplement 6—source data 1. [file elife-77779-fig2-figsupp6-data1.zip › Figure 2-figure supplement 6-source data 1/Figure 2-figure supplement 6A_rawimages/LysateBlotMyc_figure2_figuresupplement 6A.tif]

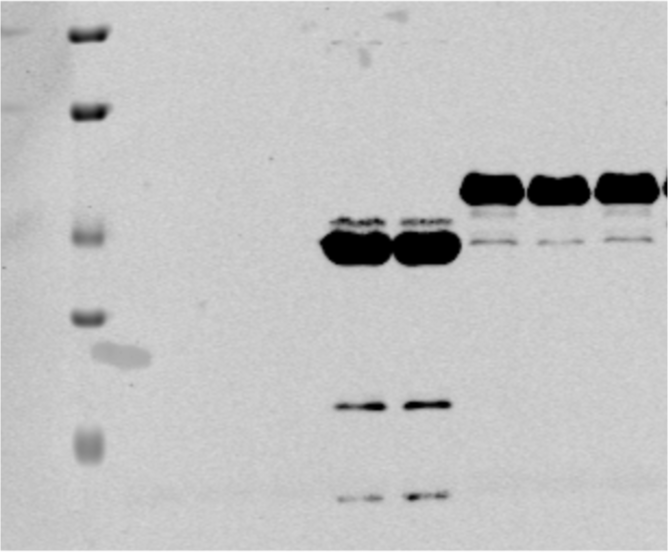

Supplement: Figure 2—figure supplement 6—source data 1. [file elife-77779-fig2-figsupp6-data1.zip › Figure 2-figure supplement 6-source data 1/Figure 2-figure supplement 6B_rawimages/Figure2_figuresupplent6B_IPOSFWBFlag.tif]

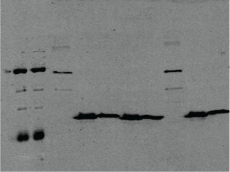

Supplement: Figure 2—figure supplement 6—source data 1. [file elife-77779-fig2-figsupp6-data1.zip › Figure 2-figure supplement 6-source data 1/Figure 2-figure supplement 6B_rawimages/Figure 2_figuresupplement 6B_LysateMyc.tif]

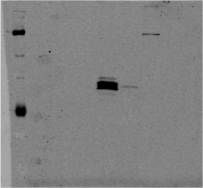

Supplement: Figure 2—figure supplement 6—source data 1. [file elife-77779-fig2-figsupp6-data1.zip › Figure 2-figure supplement 6-source data 1/Figure 2-figure supplement 6B_rawimages/Figure2_figuresupplent6B_IPOSFWBMyc.tif]

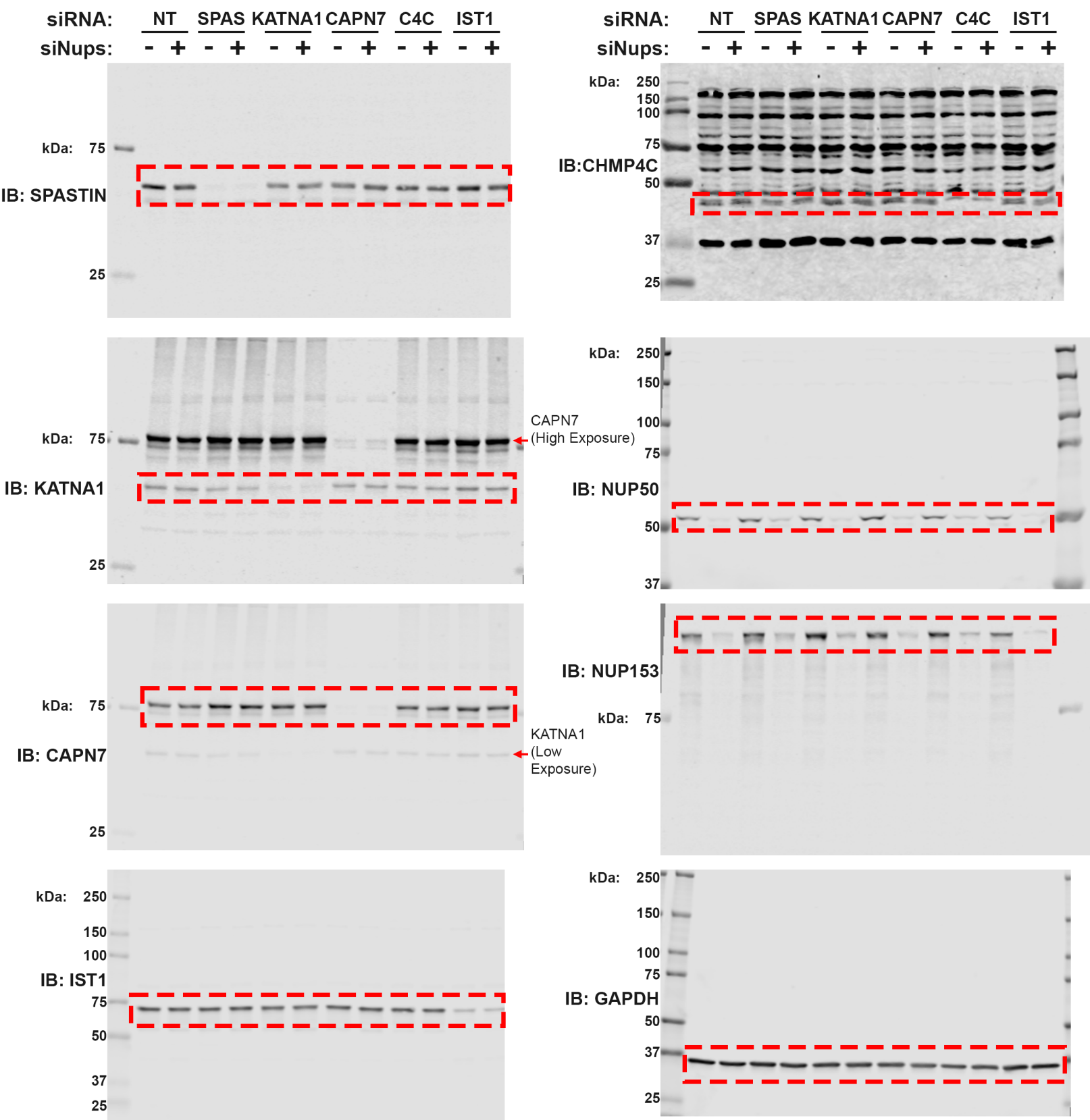

Uncropped Western blots for Figure 5 - figure supplement 1A.

Supplement: Figure 5—figure supplement 1—source data 1. [file elife-77779-fig5-figsupp1-data1.zip › Figure 5-figure supplement 1-source data 1/Figure5_figuresupplement1A_uncroppedblots.pdf]

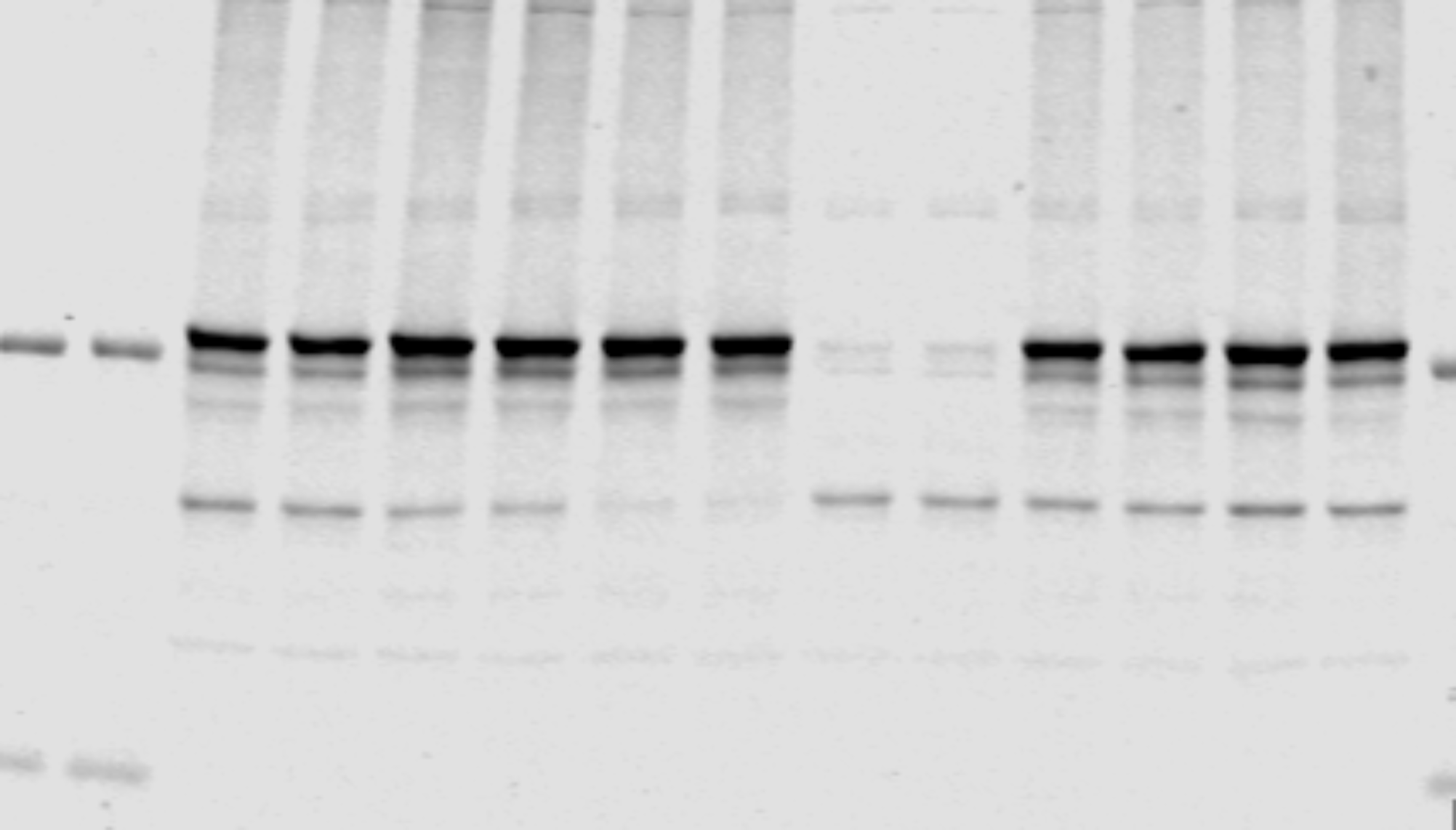

Supplement: Figure 5—figure supplement 1—source data 1. [file elife-77779-fig5-figsupp1-data1.zip › Figure 5-figure supplement 1-source data 1/Figure 5-figure supplement 1A-RawImages/CAPN7-KATNA1-high exposure.tif]

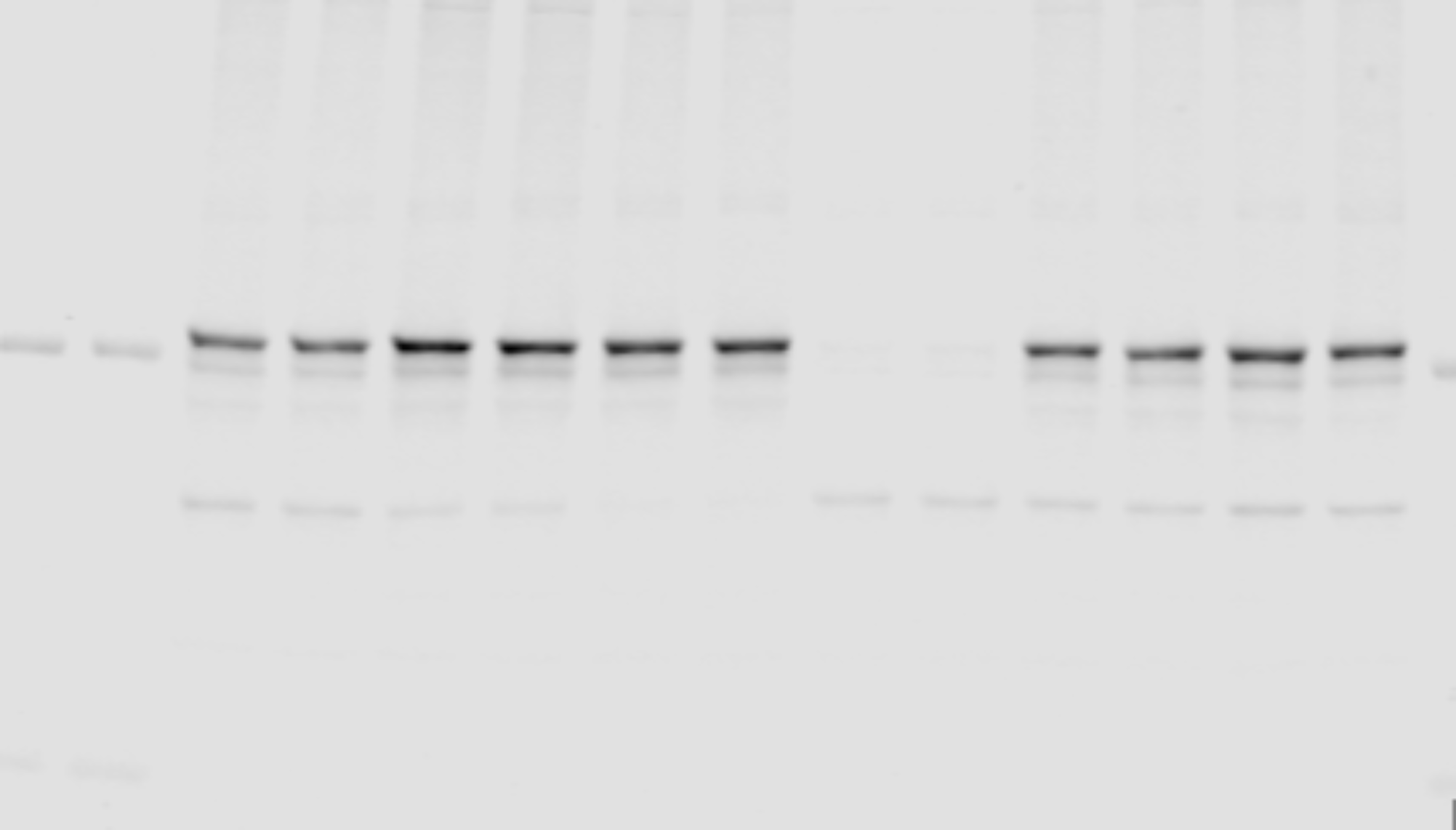

Supplement: Figure 5—figure supplement 1—source data 1. [file elife-77779-fig5-figsupp1-data1.zip › Figure 5-figure supplement 1-source data 1/Figure 5-figure supplement 1A-RawImages/CAPN7-KATNA1-Low Exposure.tif]

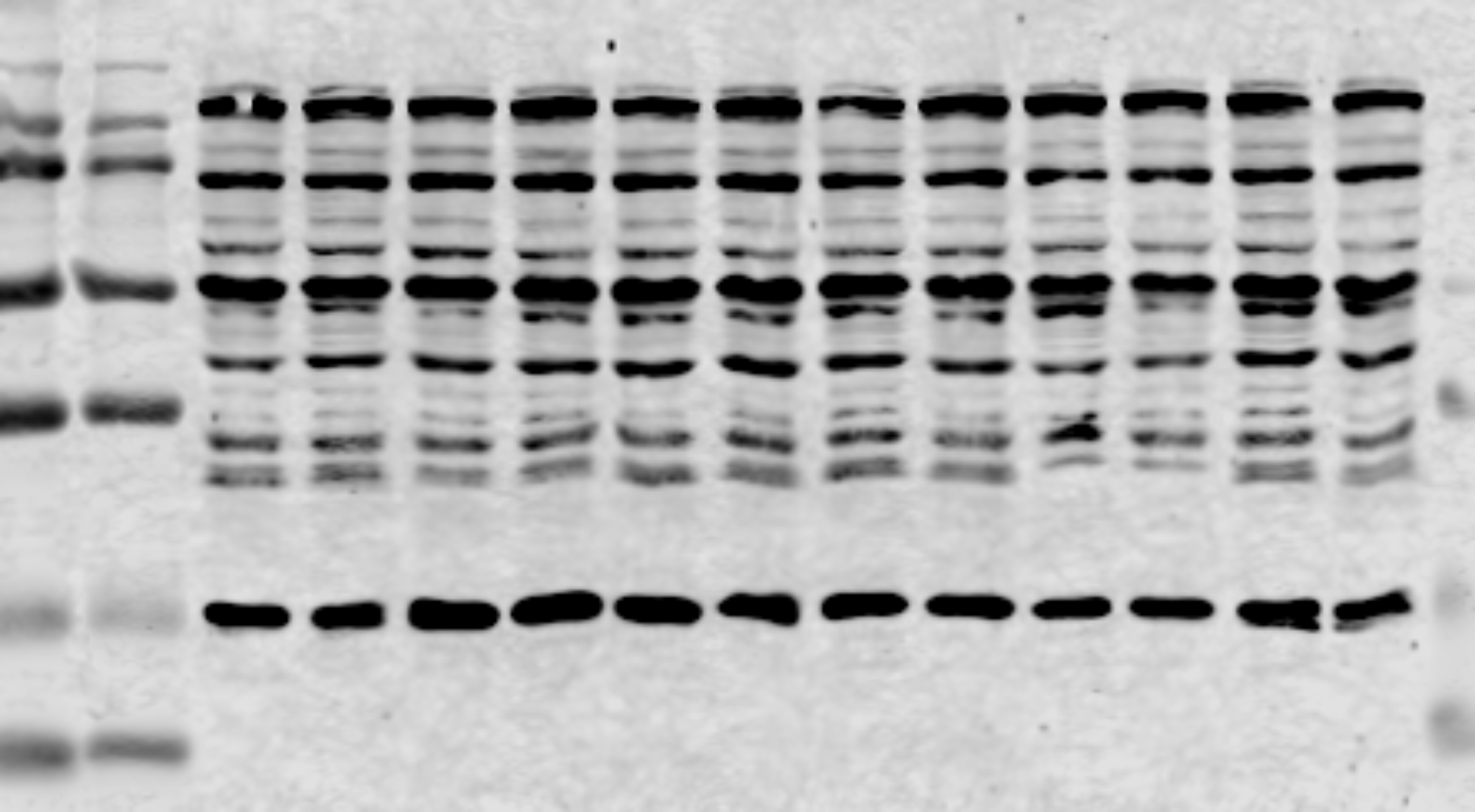

Supplement: Figure 5—figure supplement 1—source data 1. [file elife-77779-fig5-figsupp1-data1.zip › Figure 5-figure supplement 1-source data 1/Figure 5-figure supplement 1A-RawImages/CHMP4C.tif]

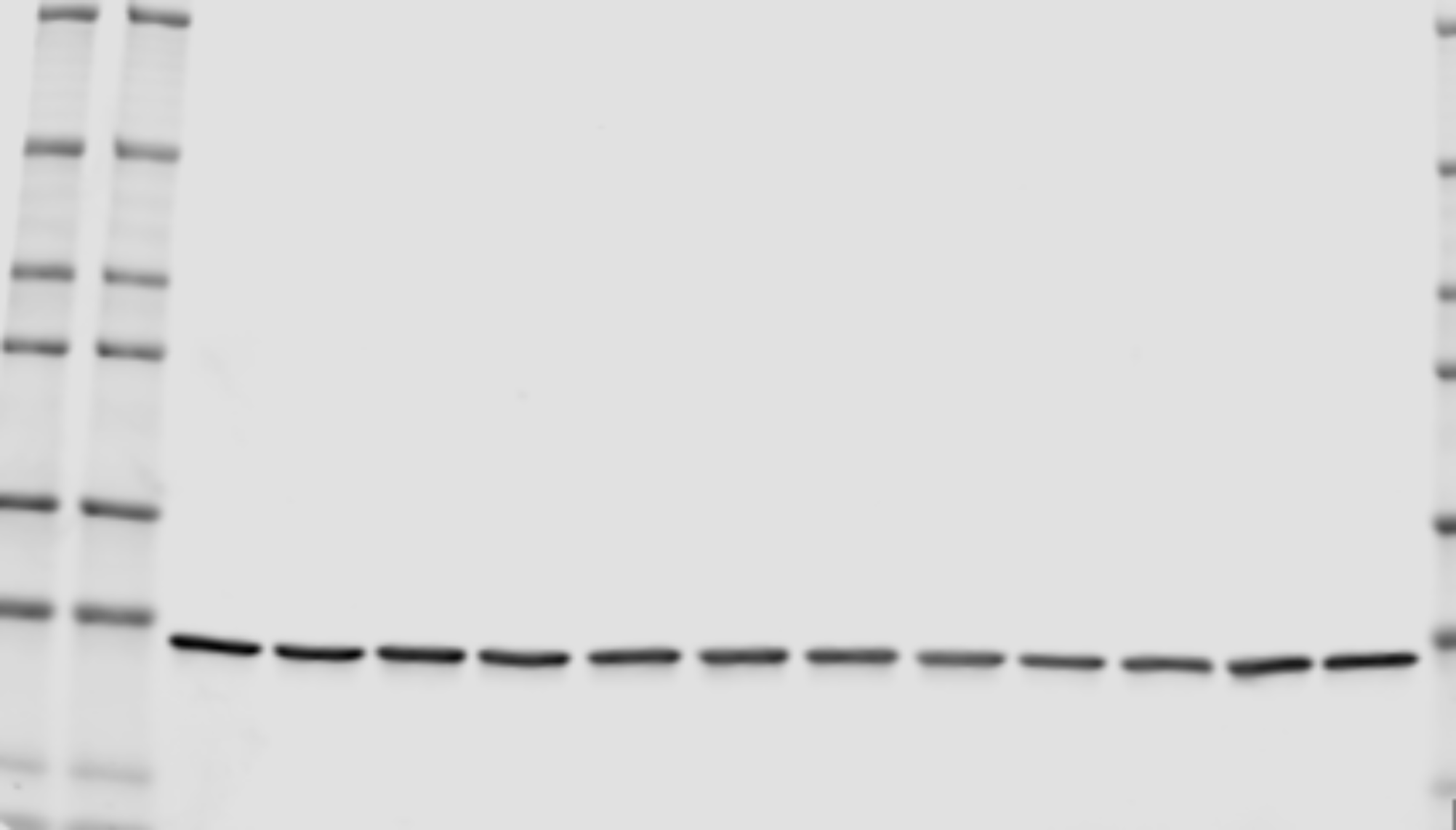

Supplement: Figure 5—figure supplement 1—source data 1. [file elife-77779-fig5-figsupp1-data1.zip › Figure 5-figure supplement 1-source data 1/Figure 5-figure supplement 1A-RawImages/GAPDH.tif]

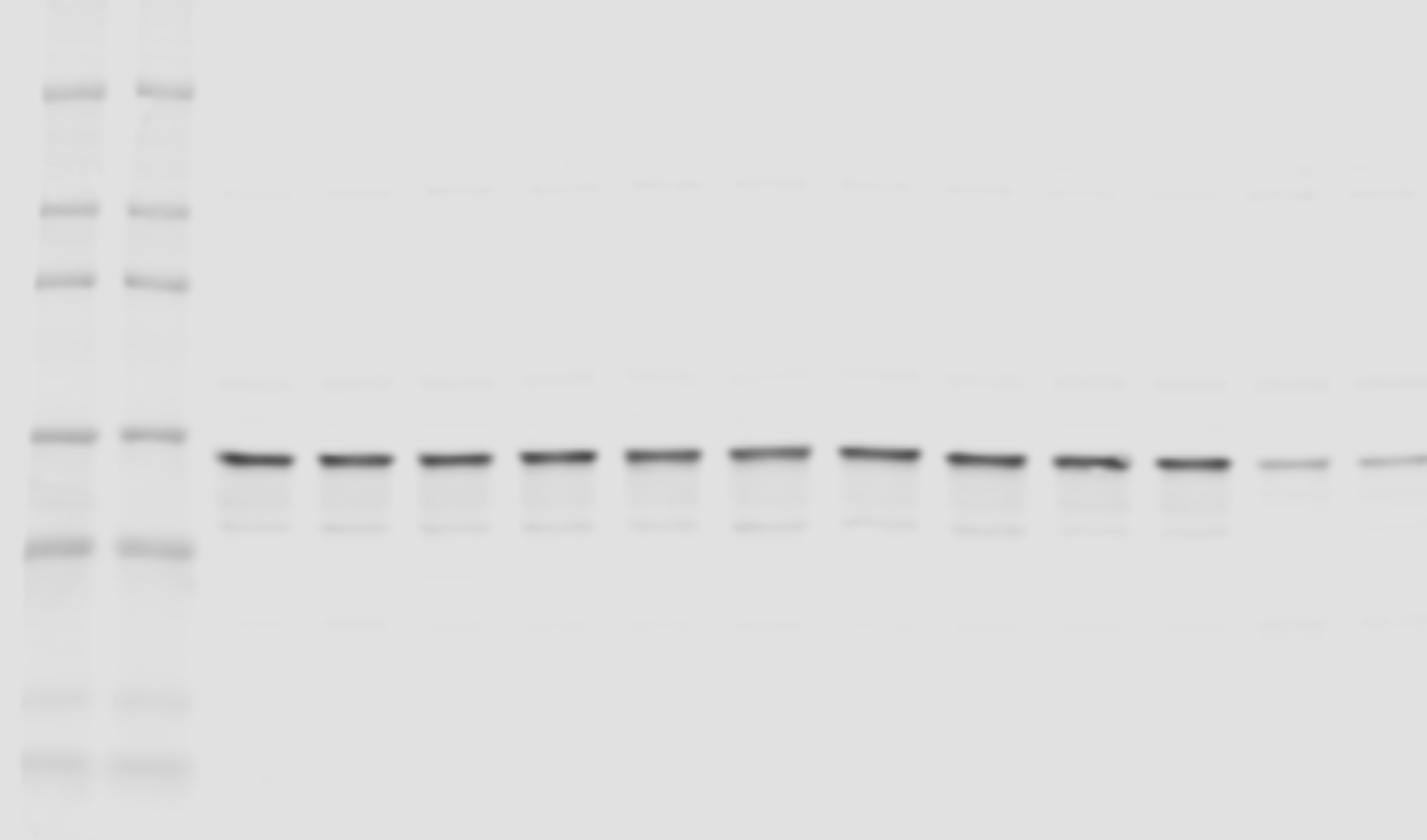

Supplement: Figure 5—figure supplement 1—source data 1. [file elife-77779-fig5-figsupp1-data1.zip › Figure 5-figure supplement 1-source data 1/Figure 5-figure supplement 1A-RawImages/IST1.tif]

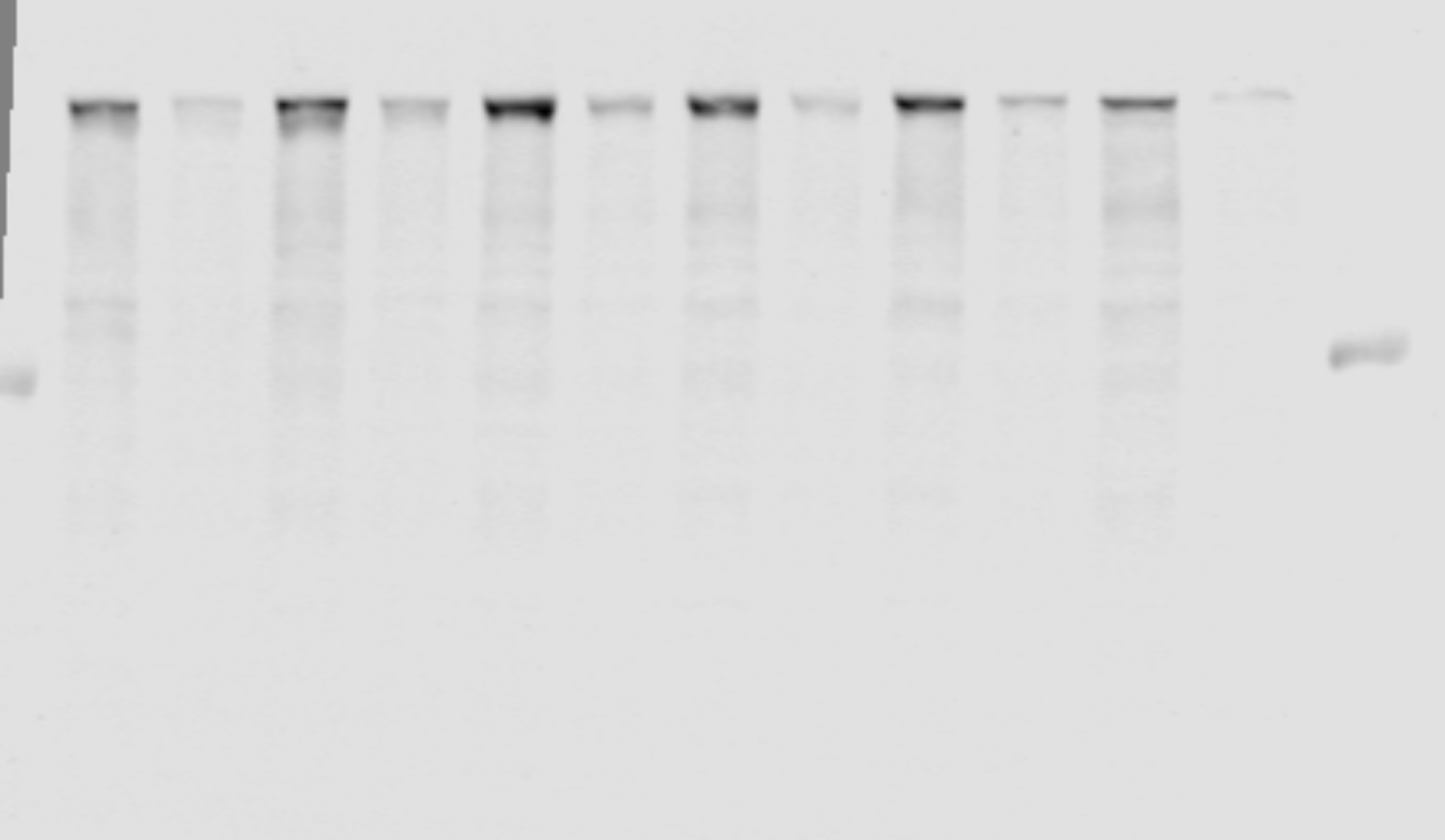

Supplement: Figure 5—figure supplement 1—source data 1. [file elife-77779-fig5-figsupp1-data1.zip › Figure 5-figure supplement 1-source data 1/Figure 5-figure supplement 1A-RawImages/NUP153.tif]

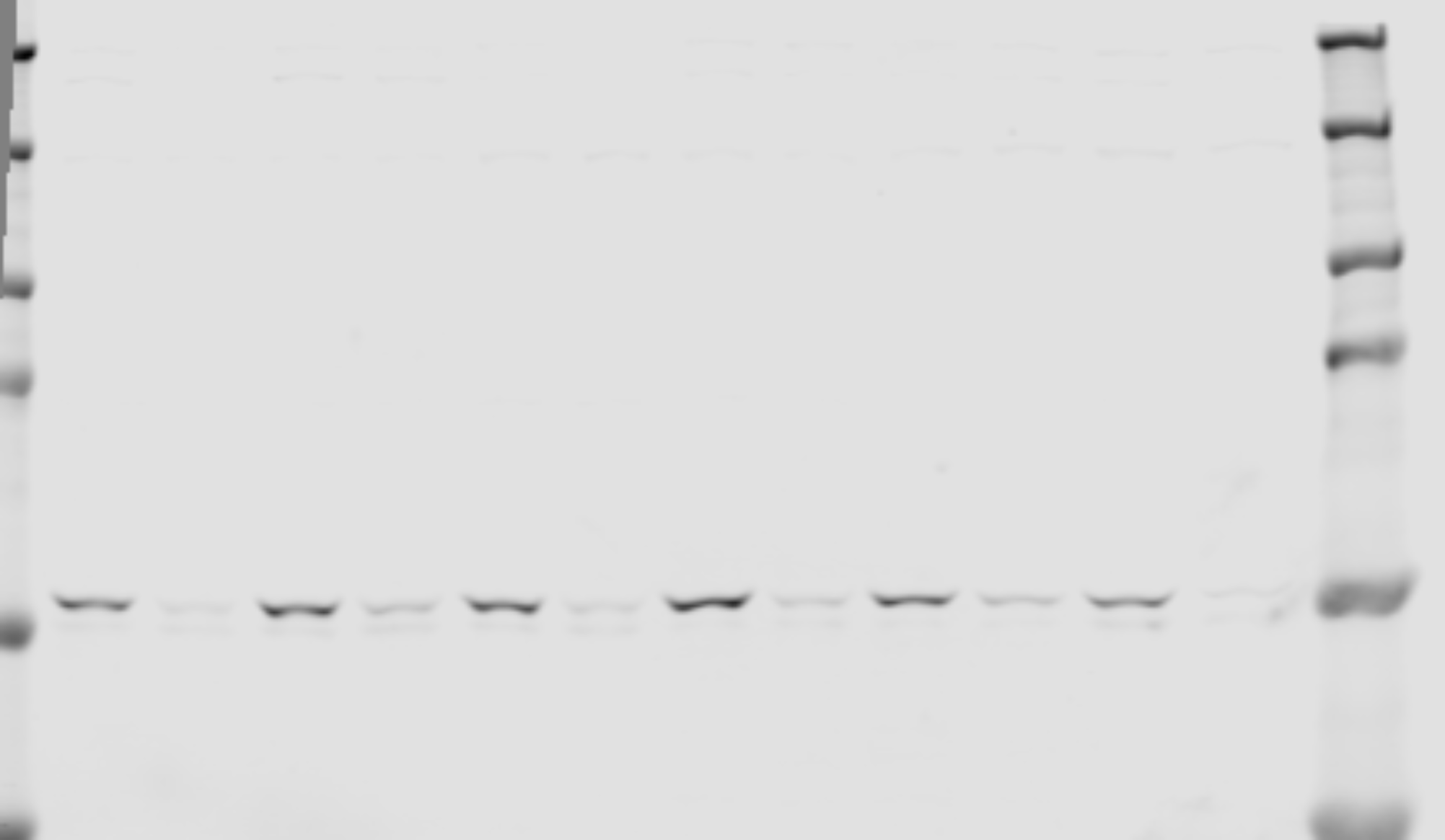

Supplement: Figure 5—figure supplement 1—source data 1. [file elife-77779-fig5-figsupp1-data1.zip › Figure 5-figure supplement 1-source data 1/Figure 5-figure supplement 1A-RawImages/NUP50.tif]

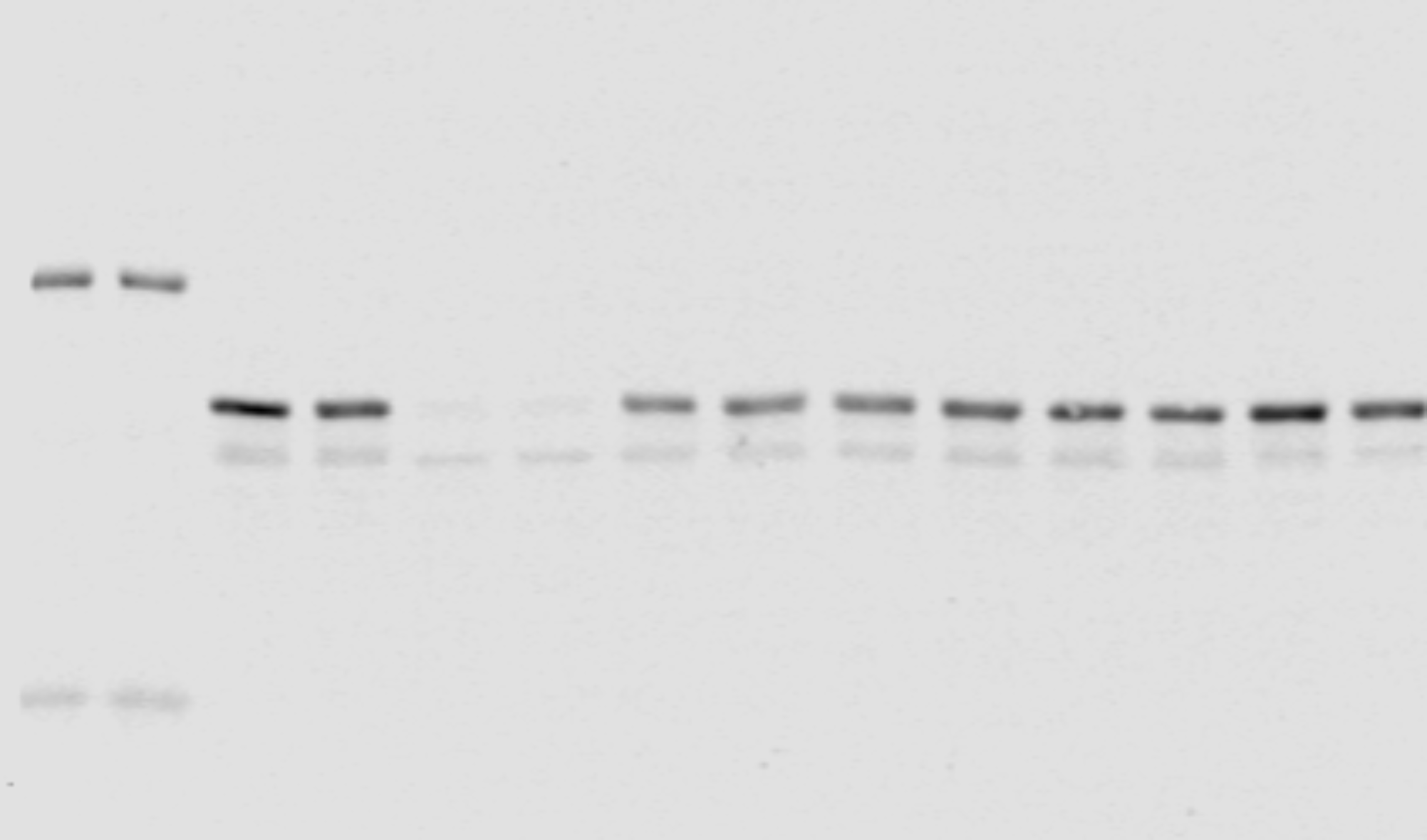

Supplement: Figure 5—figure supplement 1—source data 1. [file elife-77779-fig5-figsupp1-data1.zip › Figure 5-figure supplement 1-source data 1/Figure 5-figure supplement 1A-RawImages/SPASTIN.tif]

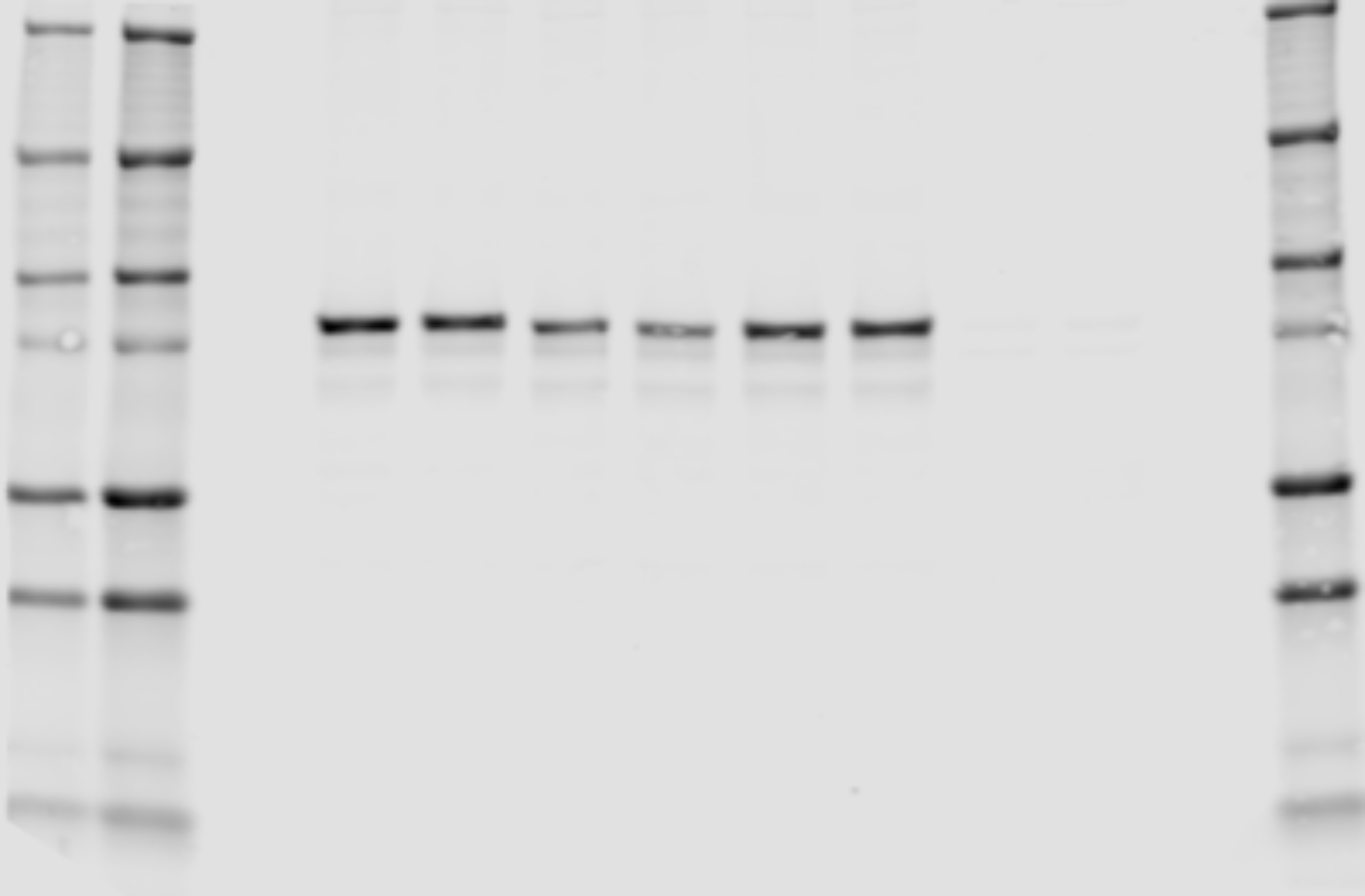

Supplement: Figure 5—figure supplement 1—source data 1. [file elife-77779-fig5-figsupp1-data1.zip › Figure 5-figure supplement 1-source data 1/Figure 5-figure supplement 1B-RawImages/CAPN7.tif]

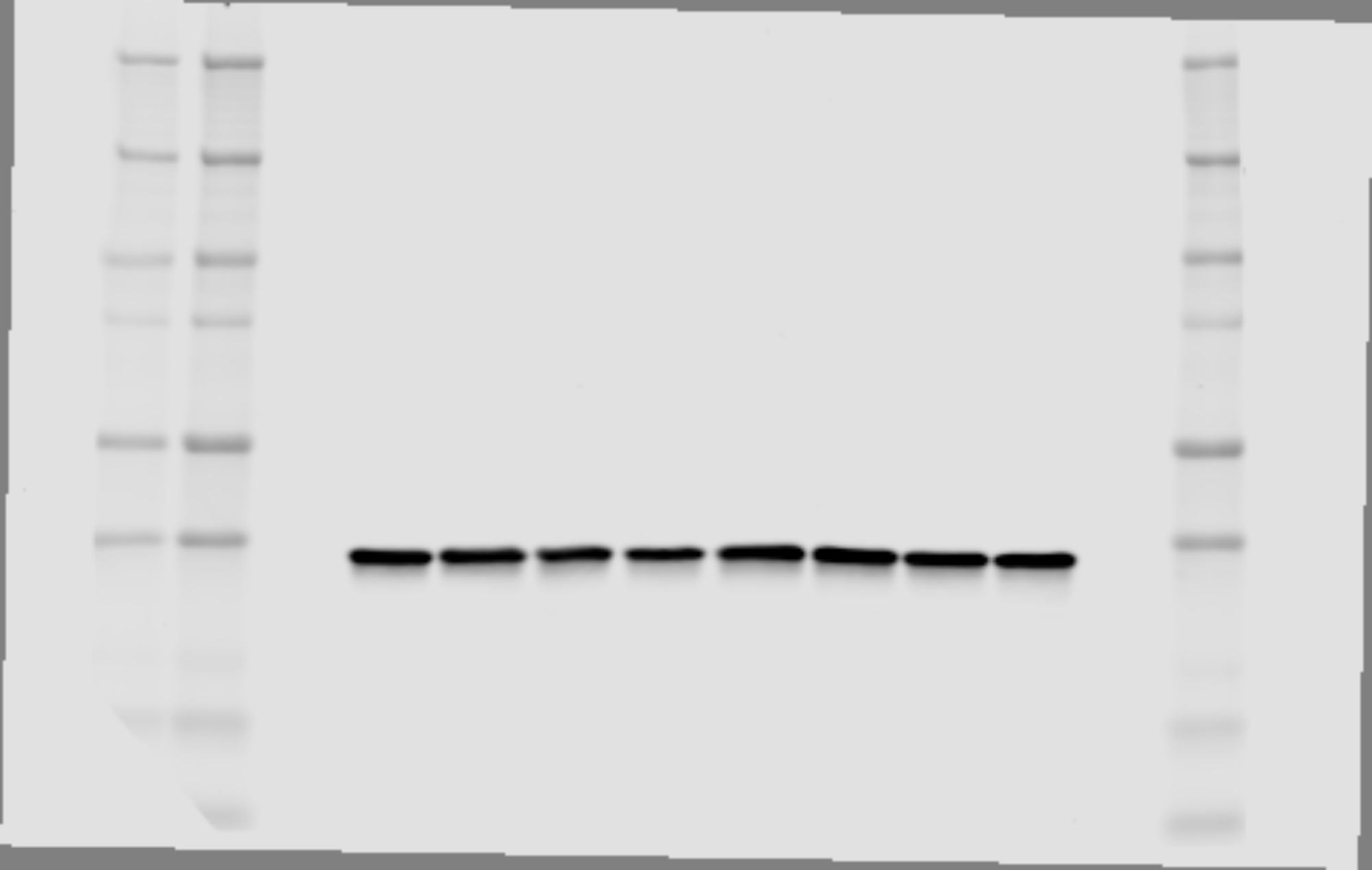

Supplement: Figure 5—figure supplement 1—source data 1. [file elife-77779-fig5-figsupp1-data1.zip › Figure 5-figure supplement 1-source data 1/Figure 5-figure supplement 1B-RawImages/GAPDH.tif]

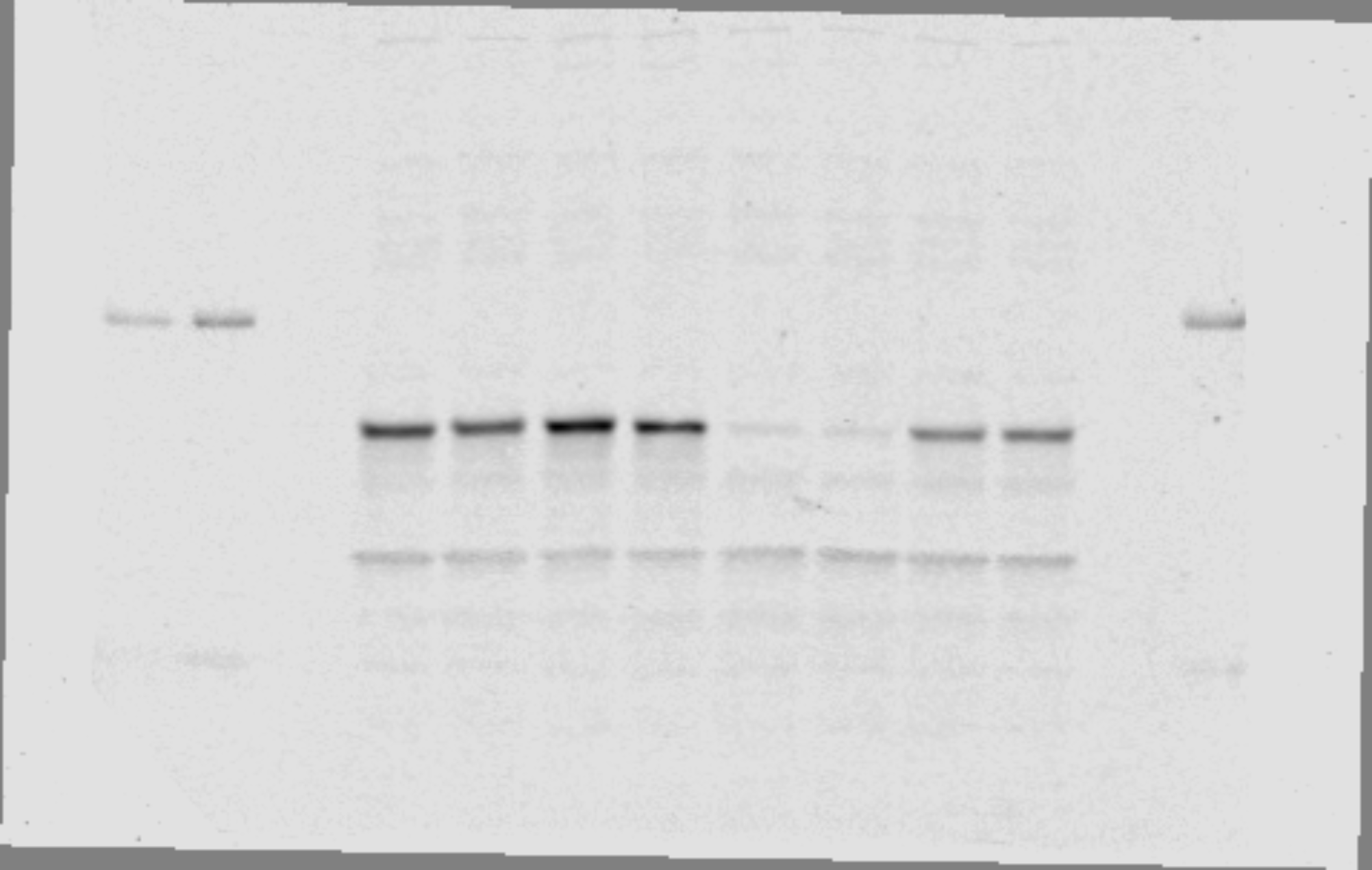

Supplement: Figure 5—figure supplement 1—source data 1. [file elife-77779-fig5-figsupp1-data1.zip › Figure 5-figure supplement 1-source data 1/Figure 5-figure supplement 1B-RawImages/KATNA1.tif]

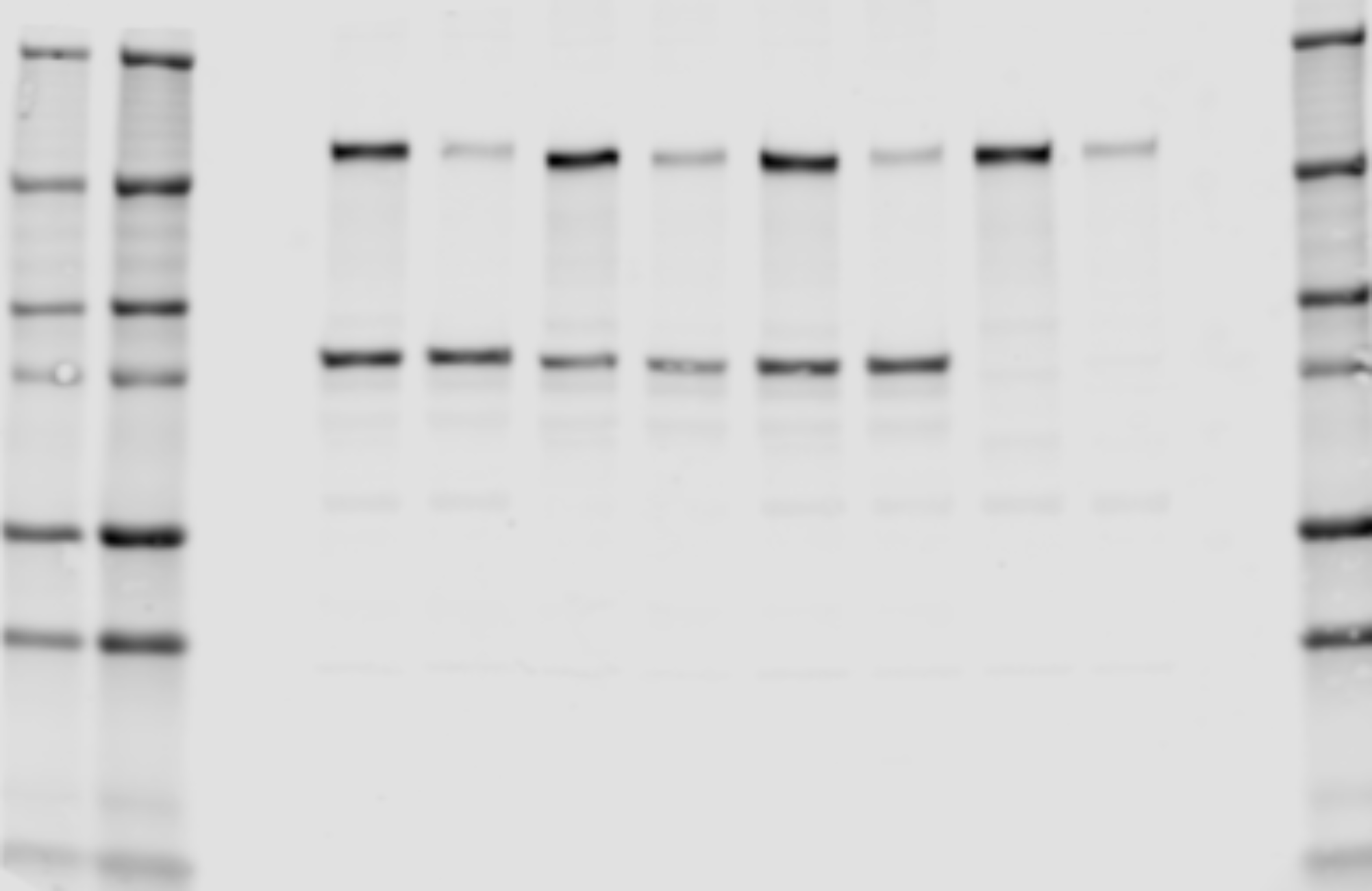

Supplement: Figure 5—figure supplement 1—source data 1. [file elife-77779-fig5-figsupp1-data1.zip › Figure 5-figure supplement 1-source data 1/Figure 5-figure supplement 1B-RawImages/Nup153-CAPN7.tif]

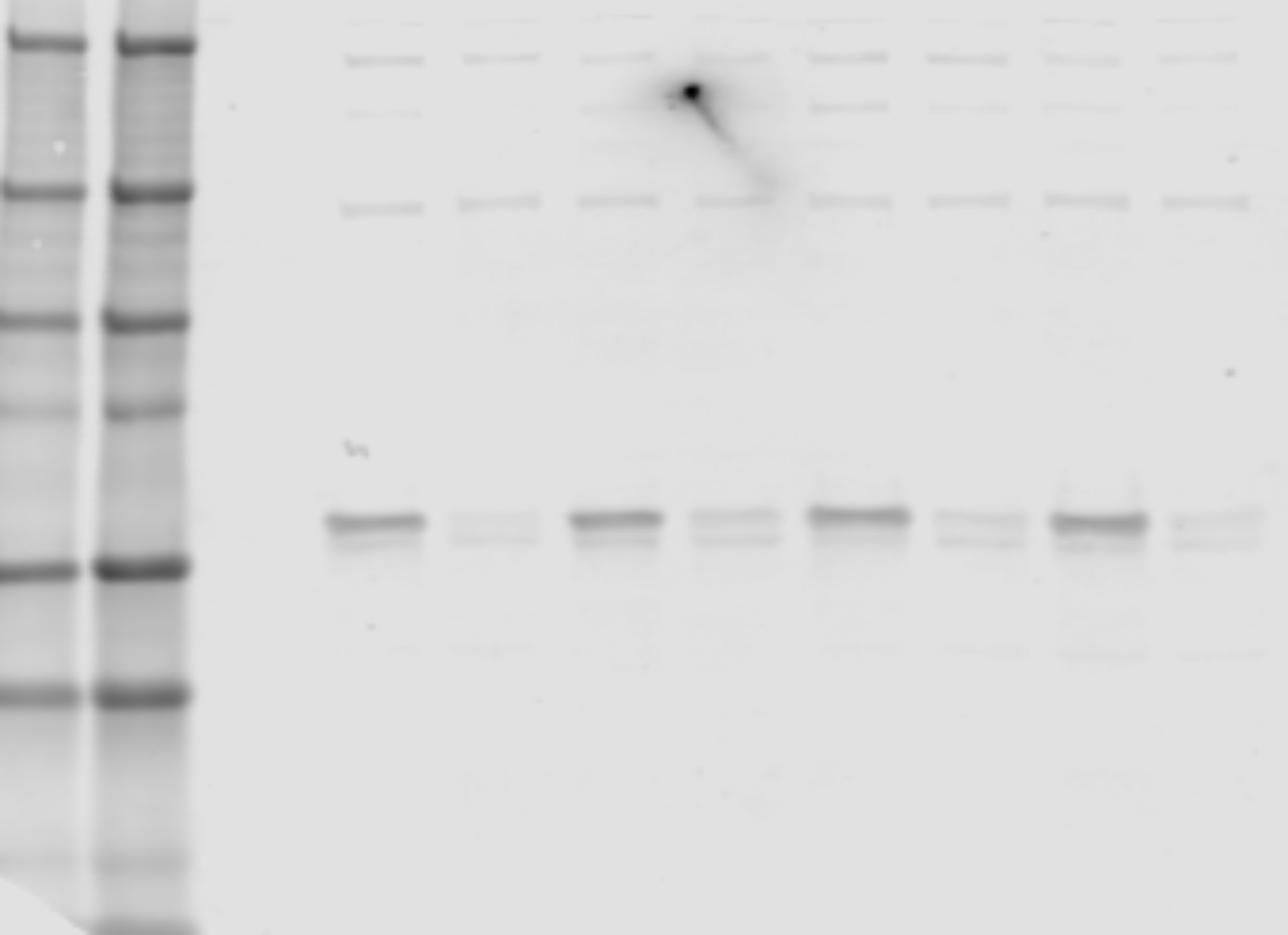

Supplement: Figure 5—figure supplement 1—source data 1. [file elife-77779-fig5-figsupp1-data1.zip › Figure 5-figure supplement 1-source data 1/Figure 5-figure supplement 1B-RawImages/NUP50.tif]

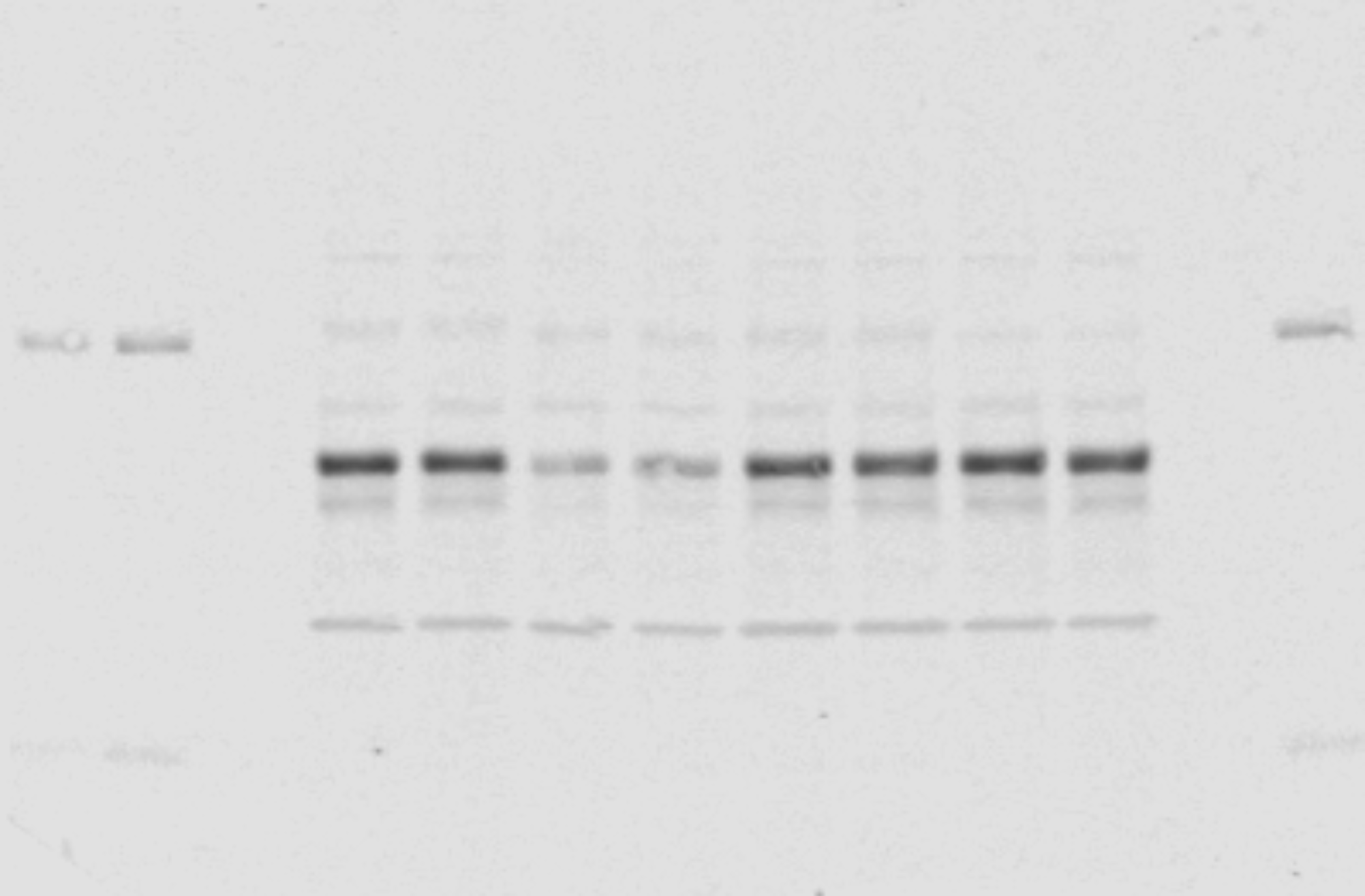

Supplement: Figure 5—figure supplement 1—source data 1. [file elife-77779-fig5-figsupp1-data1.zip › Figure 5-figure supplement 1-source data 1/Figure 5-figure supplement 1B-RawImages/SPASTIN.tif]

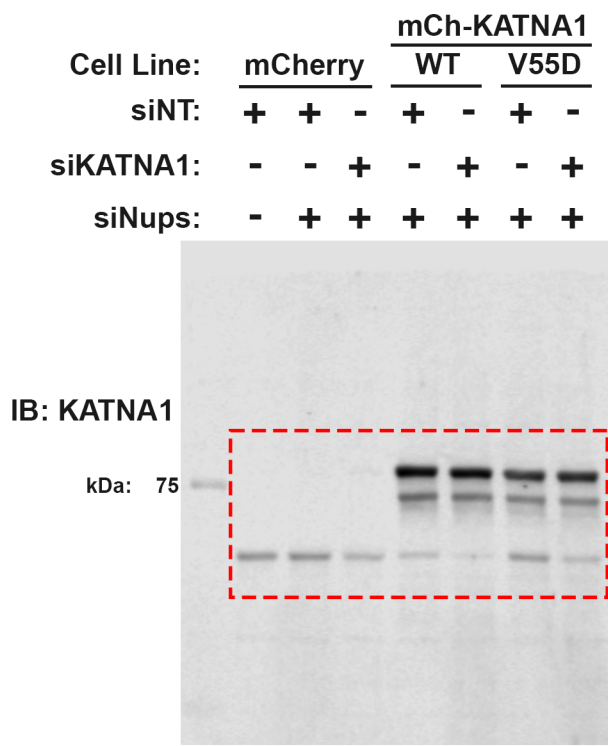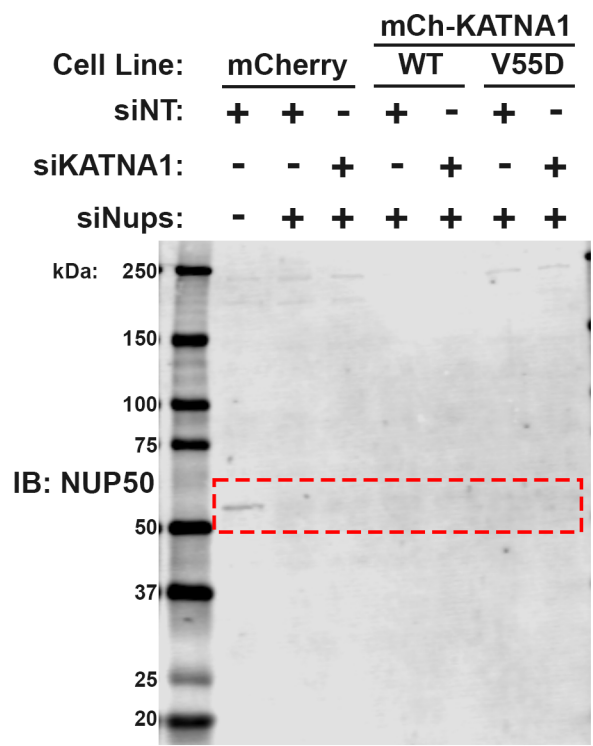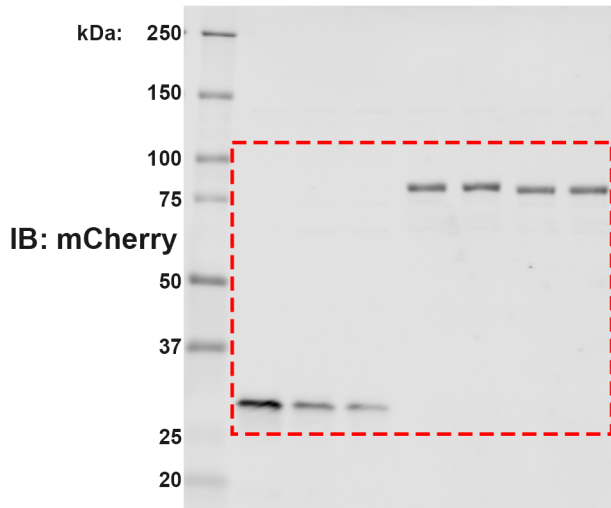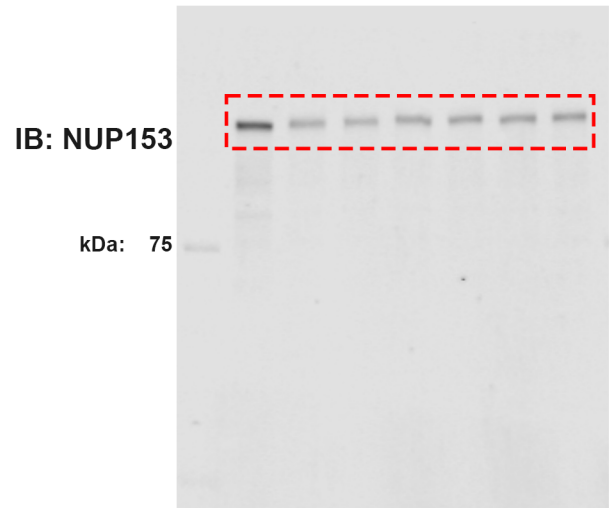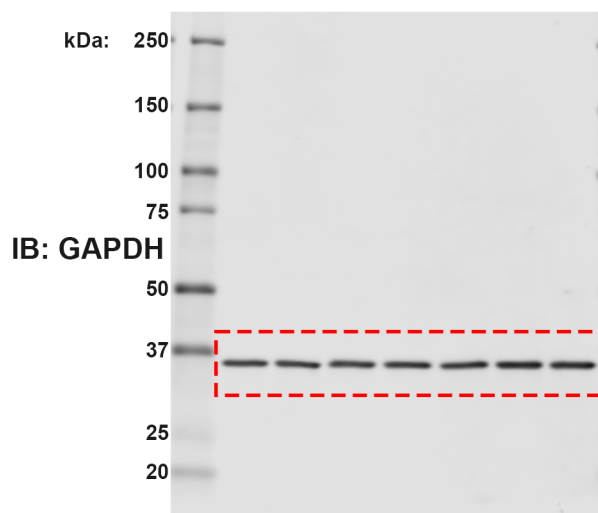

Uncropped Western blots for Figure 6-figure supplement 1B.

Supplement: Figure 6—figure supplement 1—source data 1. [file elife-77779-fig6-figsupp1-data1.zip › Figure 6-figure supplement 1 source data 1/Figure 6-figure supplement 1B uncropped blots.pdf]

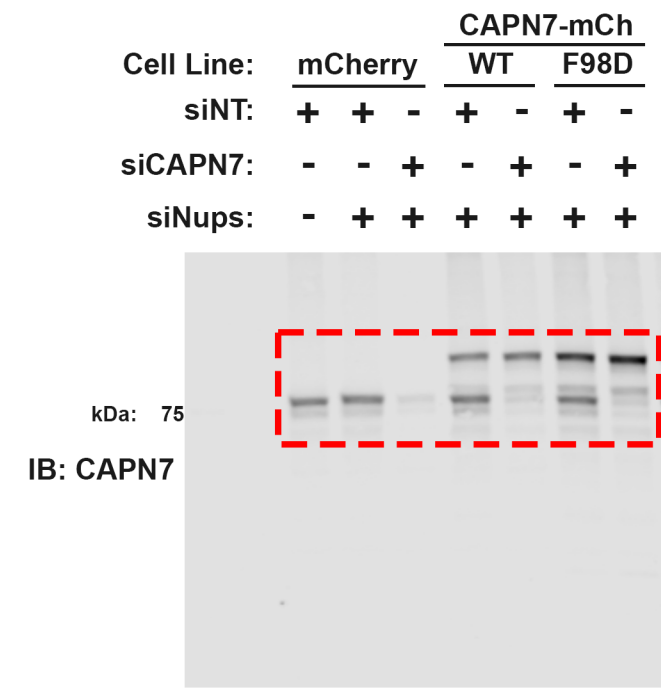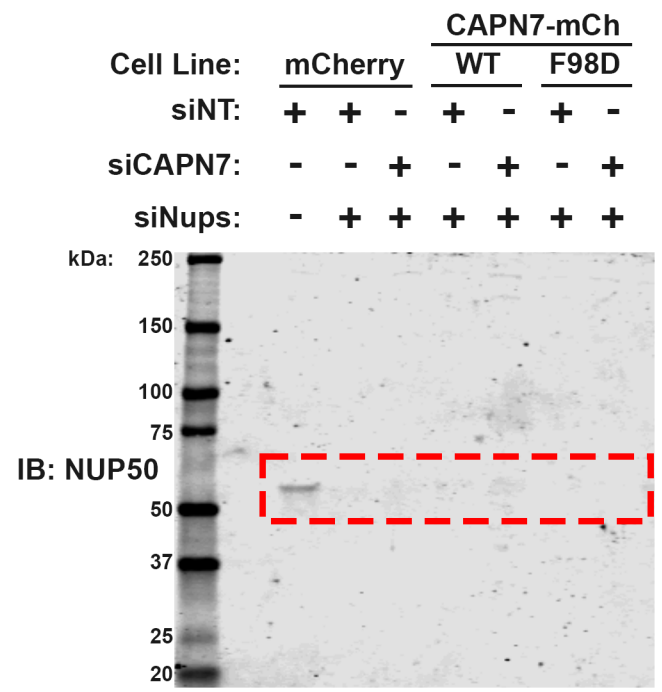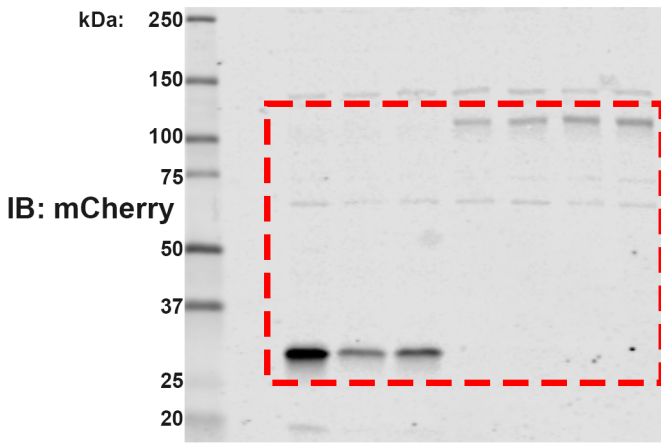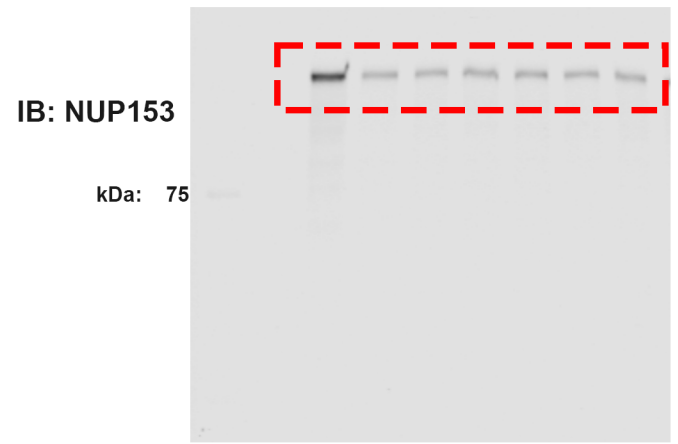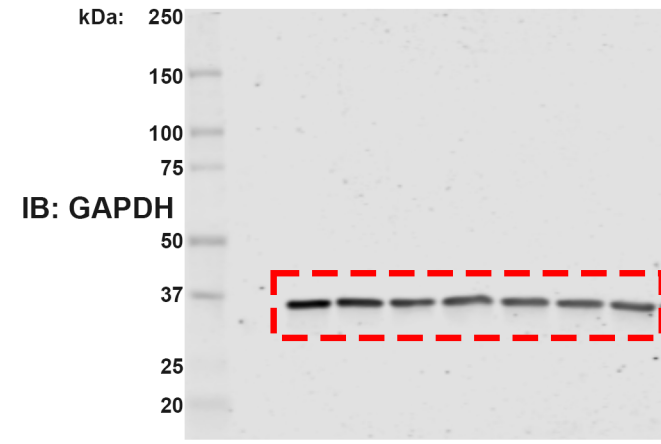

Uncropped Western blots for Figure 6-figure supplement 1C.

Supplement: Figure 6—figure supplement 1—source data 1. [file elife-77779-fig6-figsupp1-data1.zip › Figure 6-figure supplement 1 source data 1/Figure 6-figure supplement 1C uncropped blots.pdf]

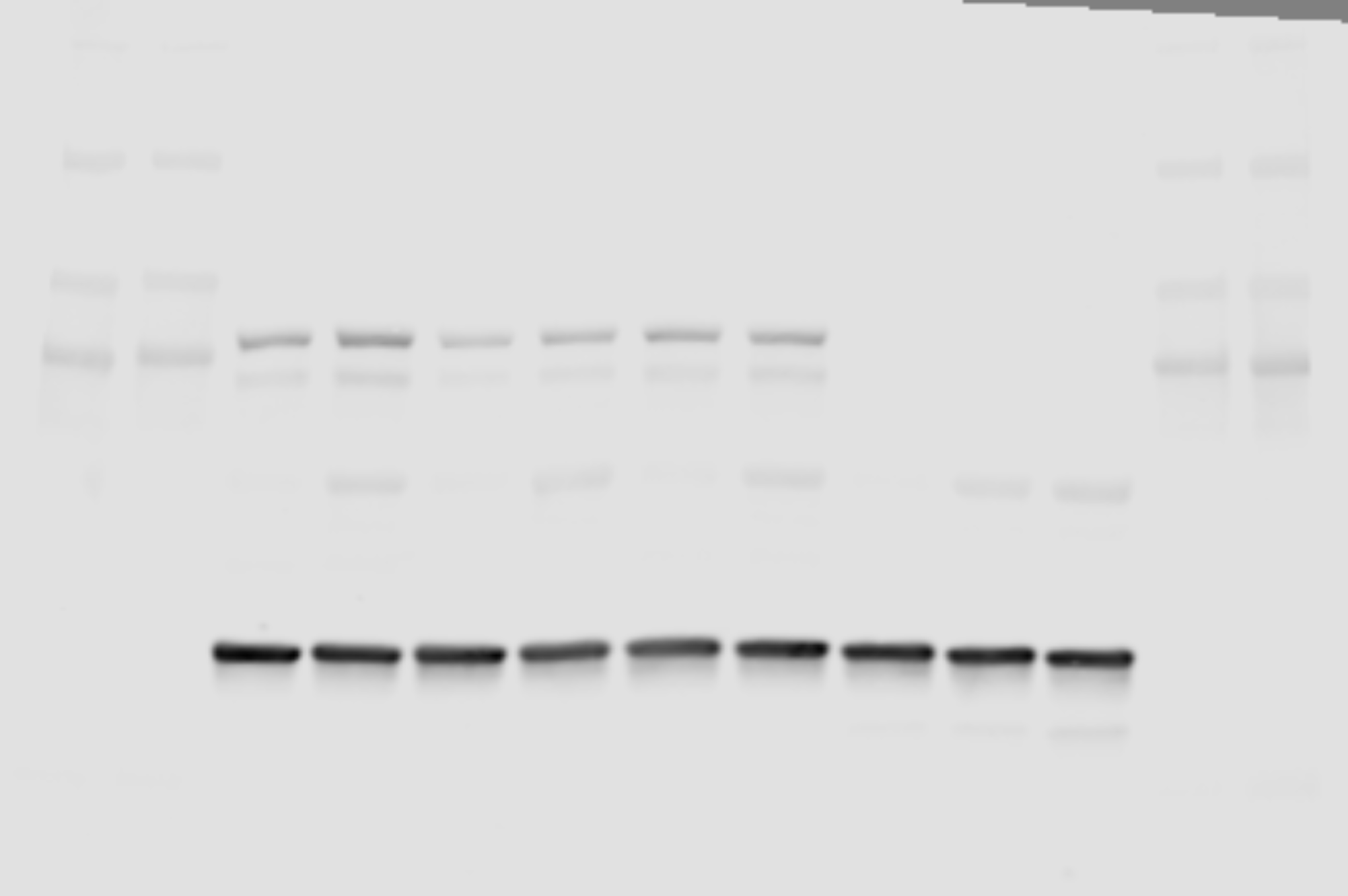

Supplement: Figure 6—figure supplement 1—source data 1. [file elife-77779-fig6-figsupp1-data1.zip › Figure 6-figure supplement 1 source data 1/Figure 6-figure supplement 1A-RawImages/GAPDH.tif]

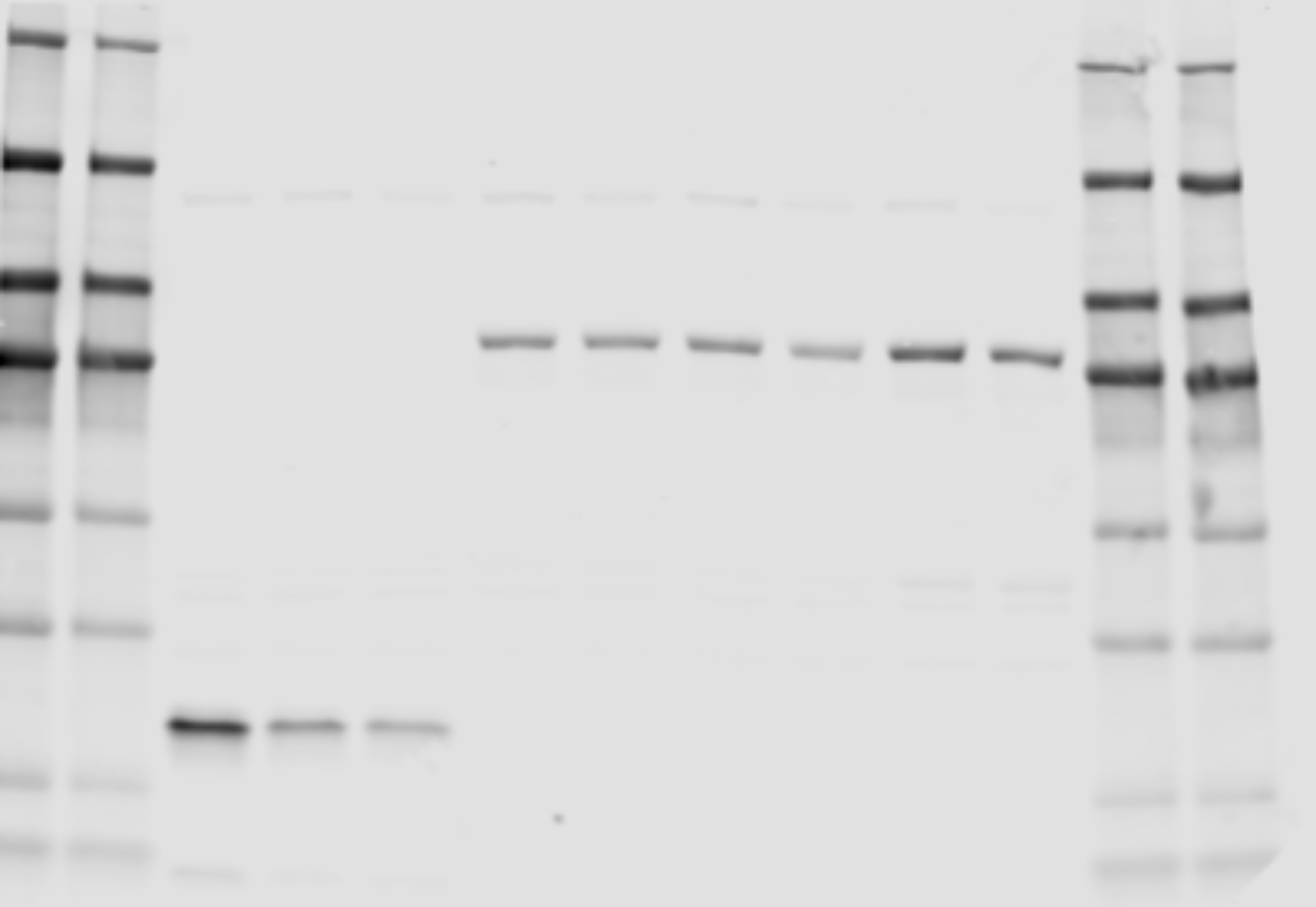

Supplement: Figure 6—figure supplement 1—source data 1. [file elife-77779-fig6-figsupp1-data1.zip › Figure 6-figure supplement 1 source data 1/Figure 6-figure supplement 1A-RawImages/mCherry.tif]

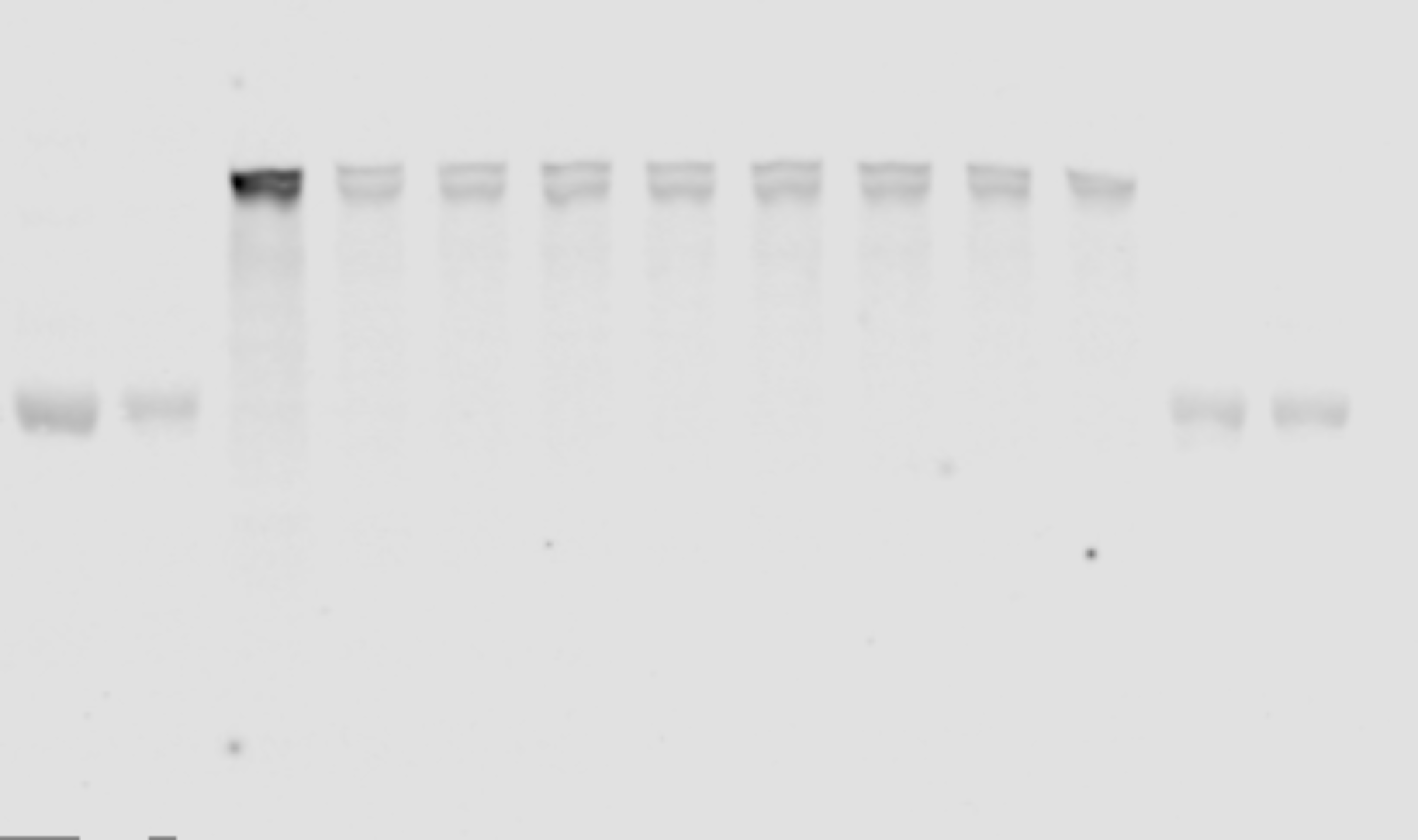

Supplement: Figure 6—figure supplement 1—source data 1. [file elife-77779-fig6-figsupp1-data1.zip › Figure 6-figure supplement 1 source data 1/Figure 6-figure supplement 1A-RawImages/NUP153.tif]

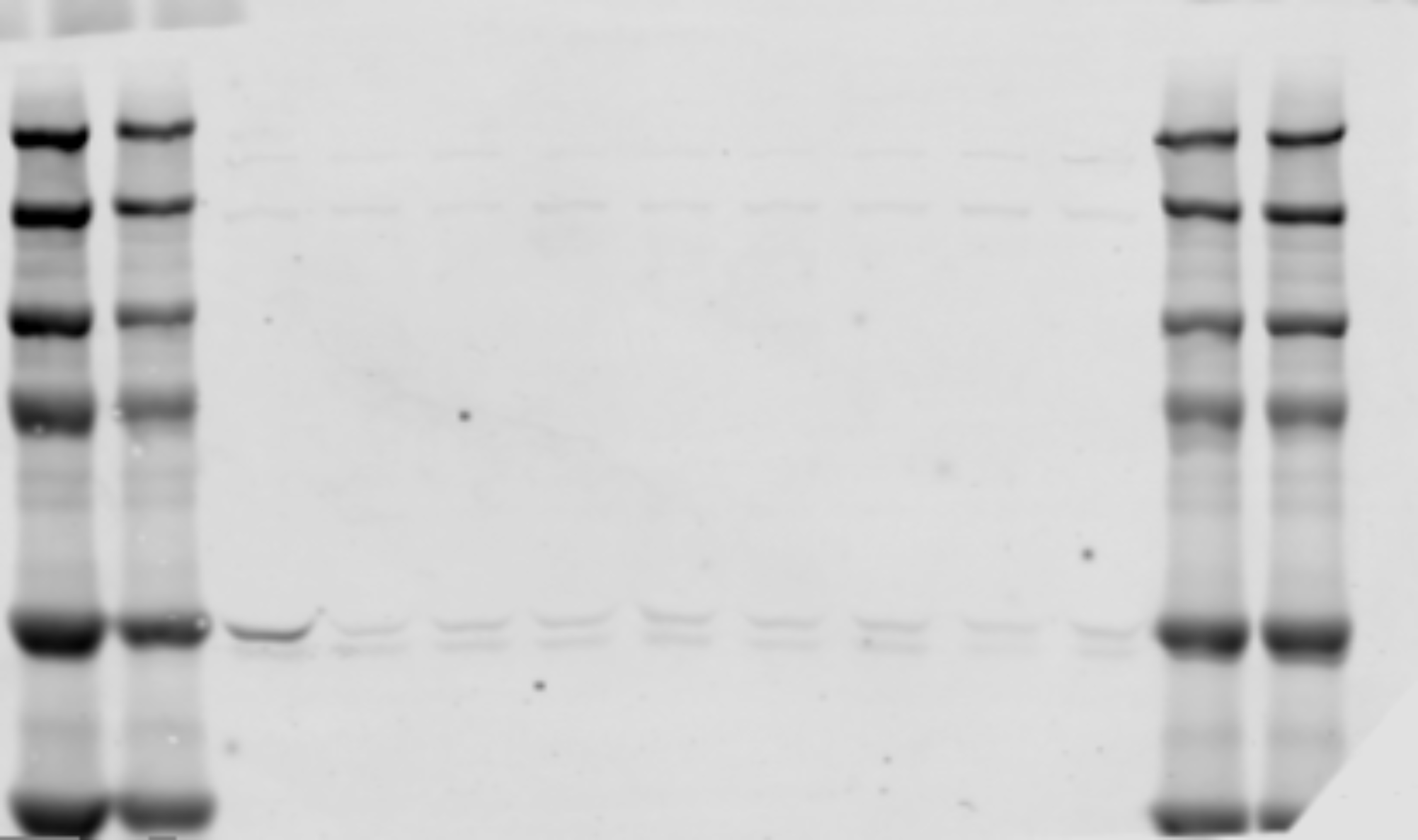

Supplement: Figure 6—figure supplement 1—source data 1. [file elife-77779-fig6-figsupp1-data1.zip › Figure 6-figure supplement 1 source data 1/Figure 6-figure supplement 1A-RawImages/NUP50.tif]

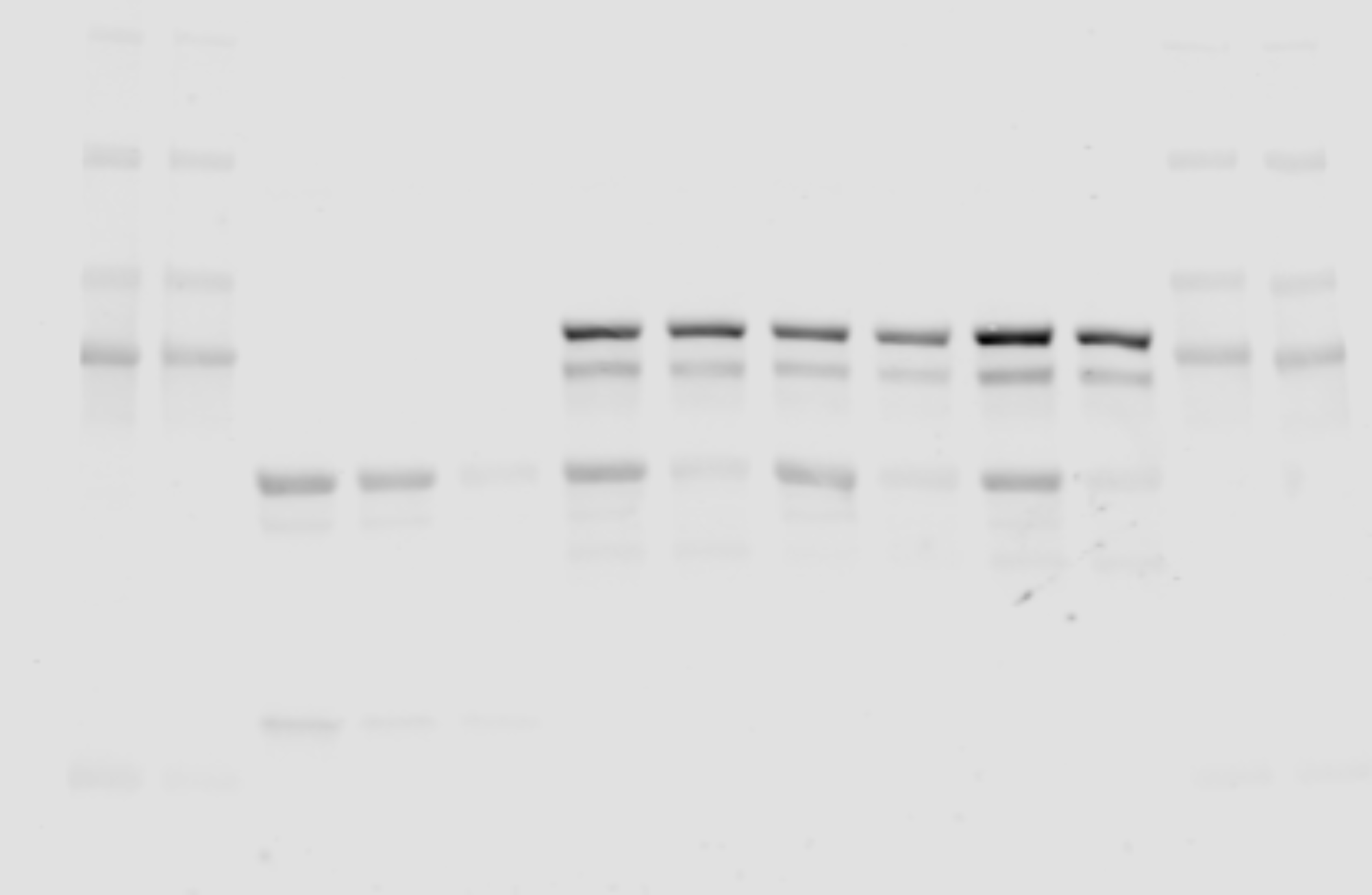

Supplement: Figure 6—figure supplement 1—source data 1. [file elife-77779-fig6-figsupp1-data1.zip › Figure 6-figure supplement 1 source data 1/Figure 6-figure supplement 1A-RawImages/SPASTIN.tif]

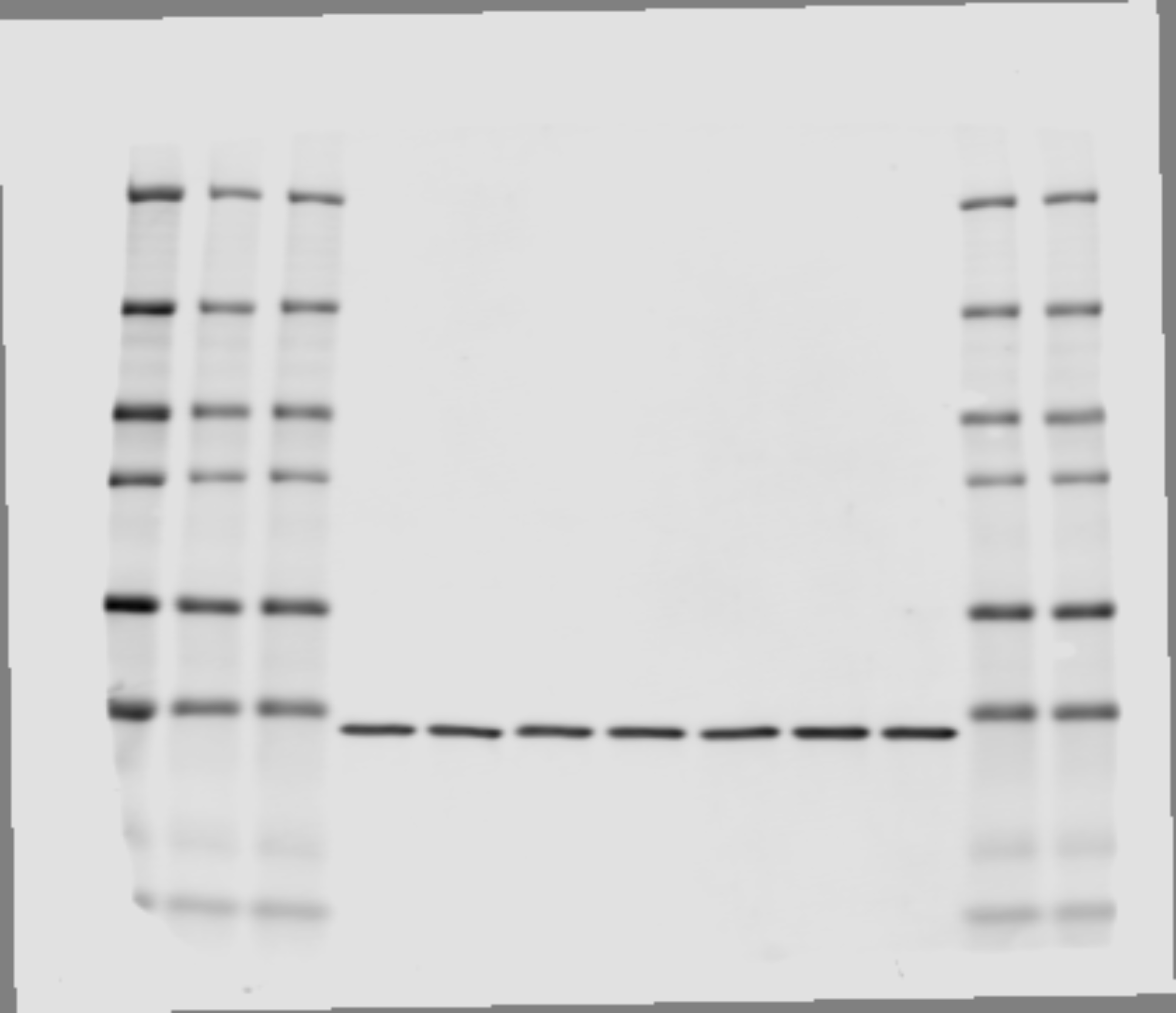

Supplement: Figure 6—figure supplement 1—source data 1. [file elife-77779-fig6-figsupp1-data1.zip › Figure 6-figure supplement 1 source data 1/Figure 6-figure supplement 1B-RawImages/GAPDH.tif]

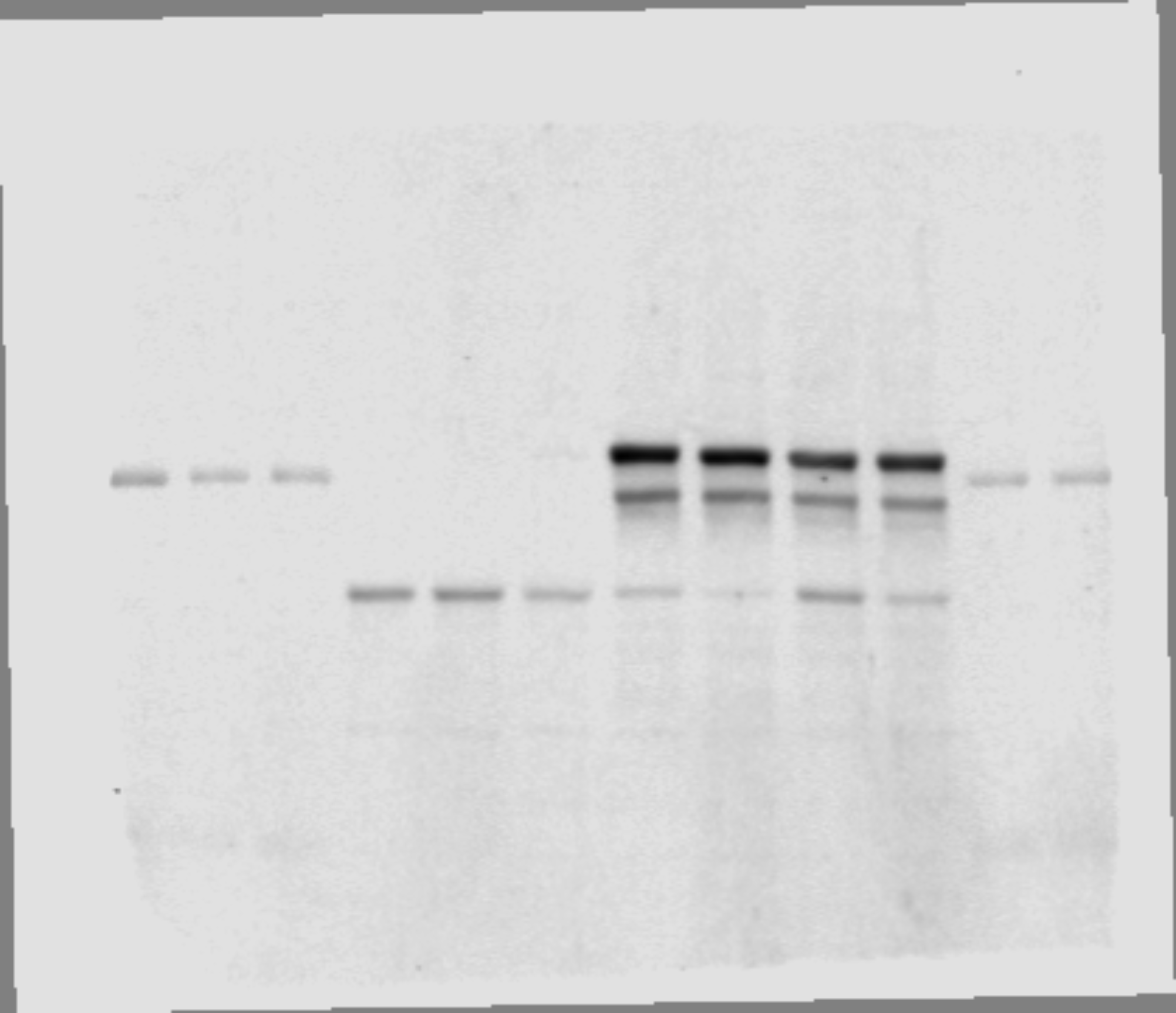

Supplement: Figure 6—figure supplement 1—source data 1. [file elife-77779-fig6-figsupp1-data1.zip › Figure 6-figure supplement 1 source data 1/Figure 6-figure supplement 1B-RawImages/KATNA1.tif]

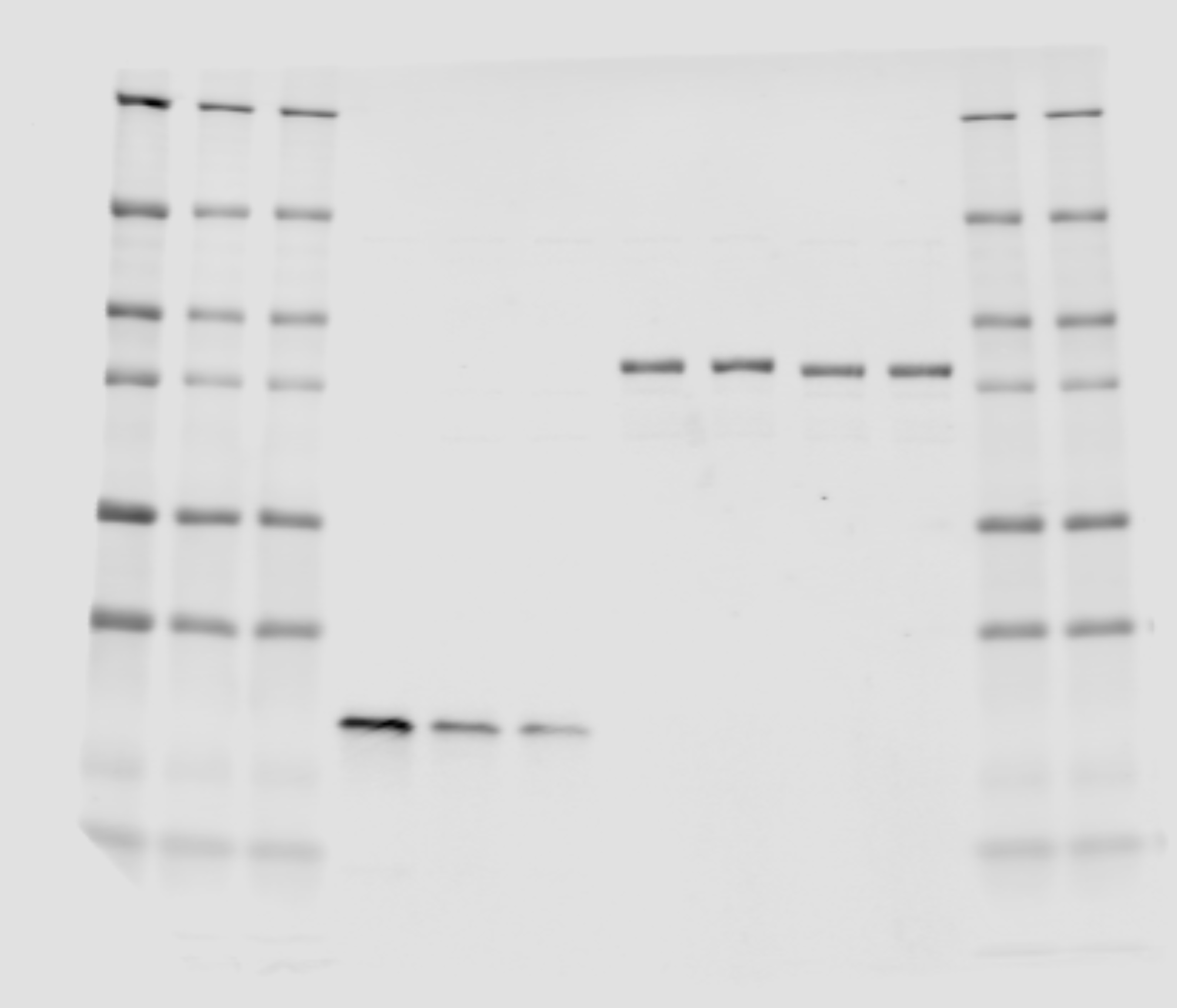

Supplement: Figure 6—figure supplement 1—source data 1. [file elife-77779-fig6-figsupp1-data1.zip › Figure 6-figure supplement 1 source data 1/Figure 6-figure supplement 1B-RawImages/mCherry.tif]

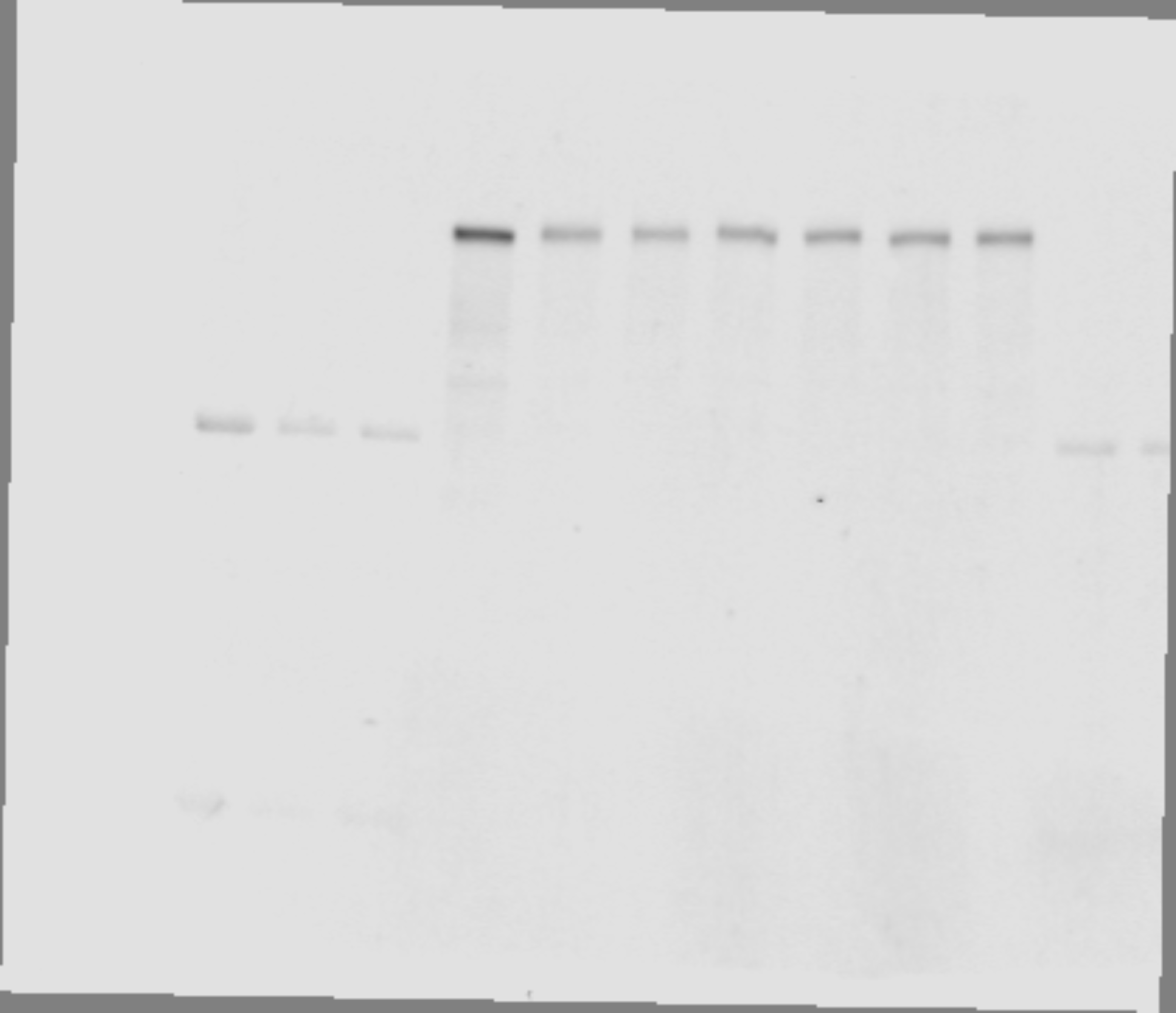

Supplement: Figure 6—figure supplement 1—source data 1. [file elife-77779-fig6-figsupp1-data1.zip › Figure 6-figure supplement 1 source data 1/Figure 6-figure supplement 1B-RawImages/NUP153.tif]

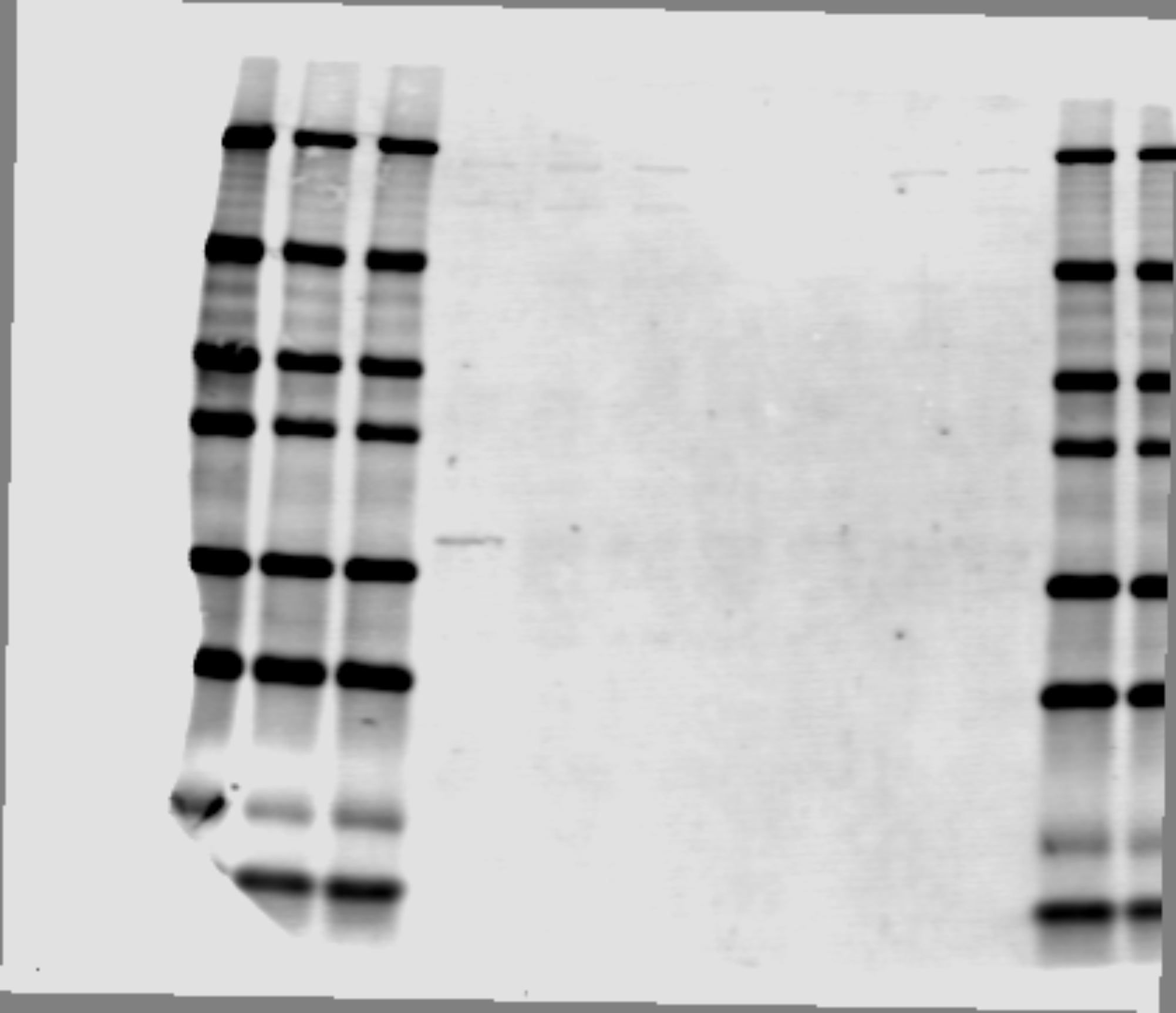

Supplement: Figure 6—figure supplement 1—source data 1. [file elife-77779-fig6-figsupp1-data1.zip › Figure 6-figure supplement 1 source data 1/Figure 6-figure supplement 1B-RawImages/NUP50.tif]

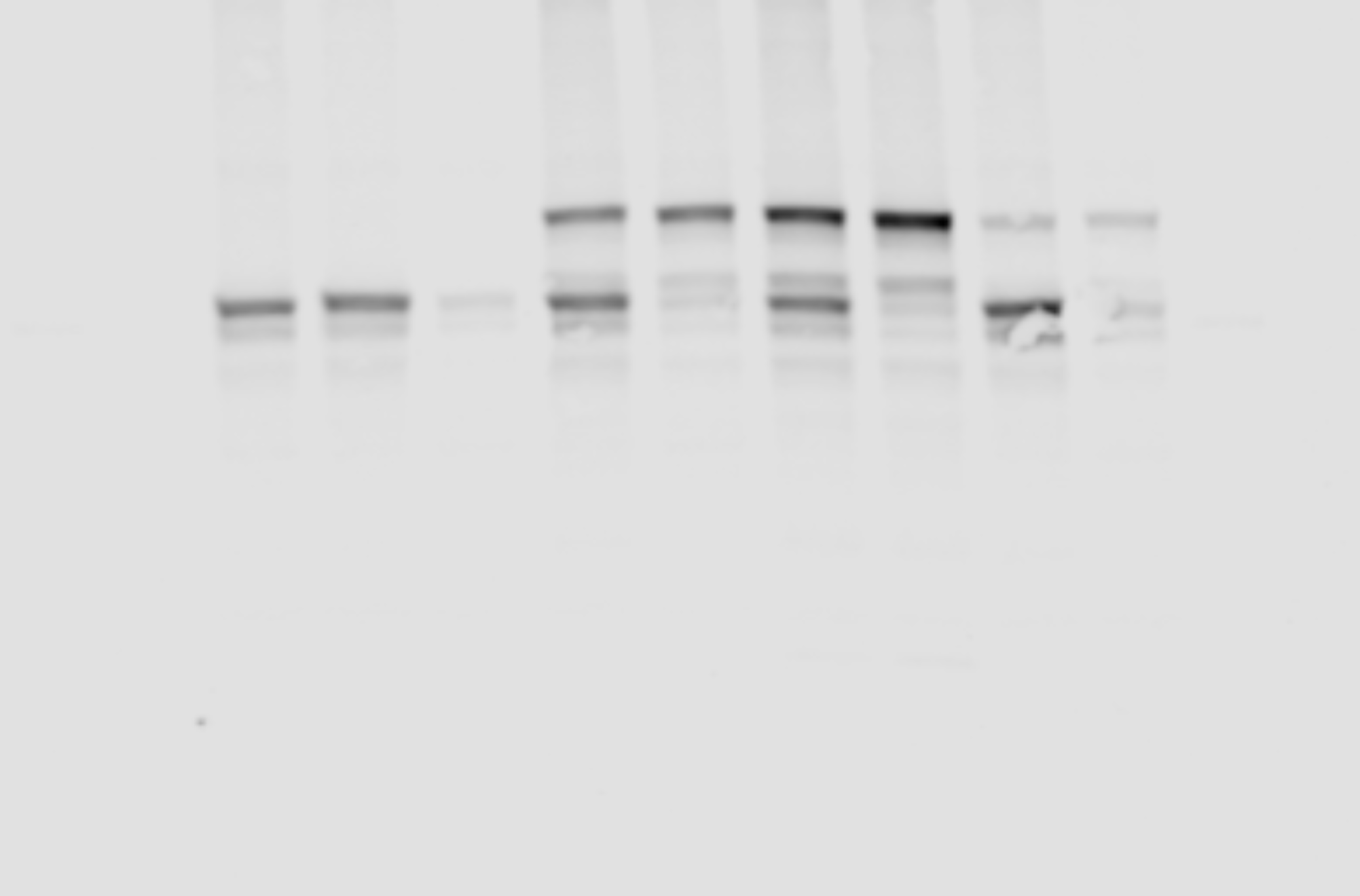

Supplement: Figure 6—figure supplement 1—source data 1. [file elife-77779-fig6-figsupp1-data1.zip › Figure 6-figure supplement 1 source data 1/Figure 6-figure supplement 1C-RawImages/CAPN7.tif]

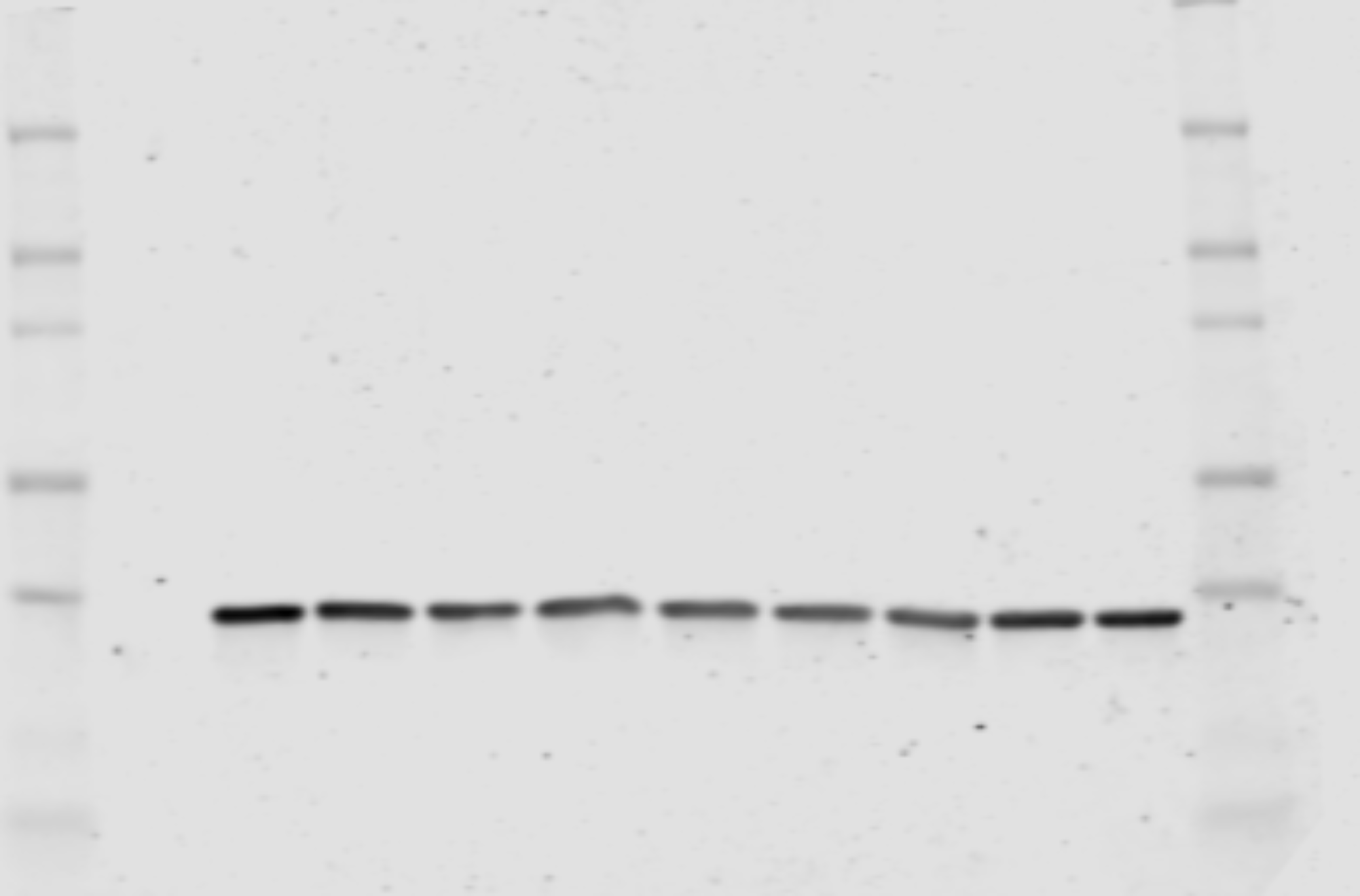

Supplement: Figure 6—figure supplement 1—source data 1. [file elife-77779-fig6-figsupp1-data1.zip › Figure 6-figure supplement 1 source data 1/Figure 6-figure supplement 1C-RawImages/GAPDH.tif]

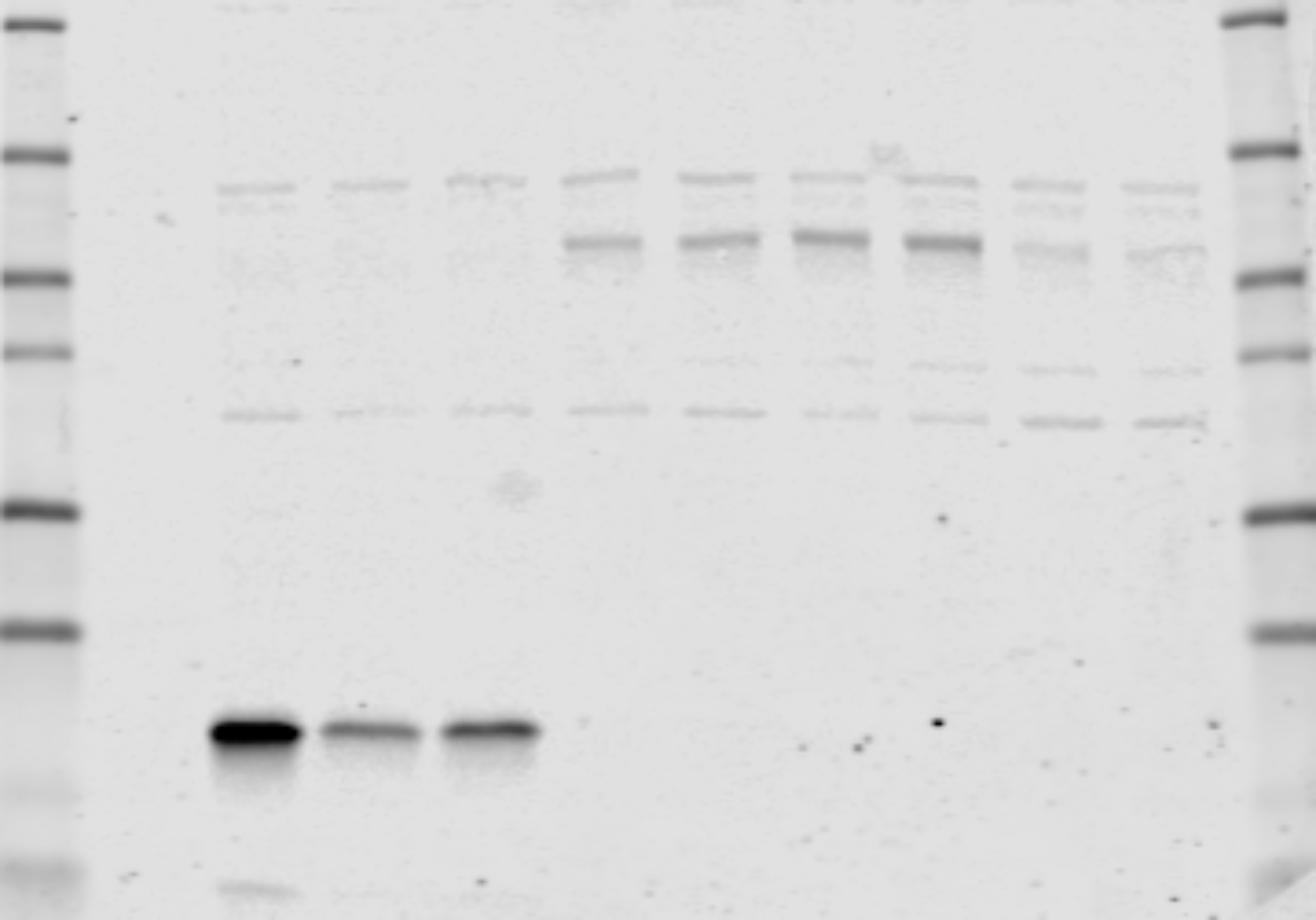

Supplement: Figure 6—figure supplement 1—source data 1. [file elife-77779-fig6-figsupp1-data1.zip › Figure 6-figure supplement 1 source data 1/Figure 6-figure supplement 1C-RawImages/mCherry.tif]

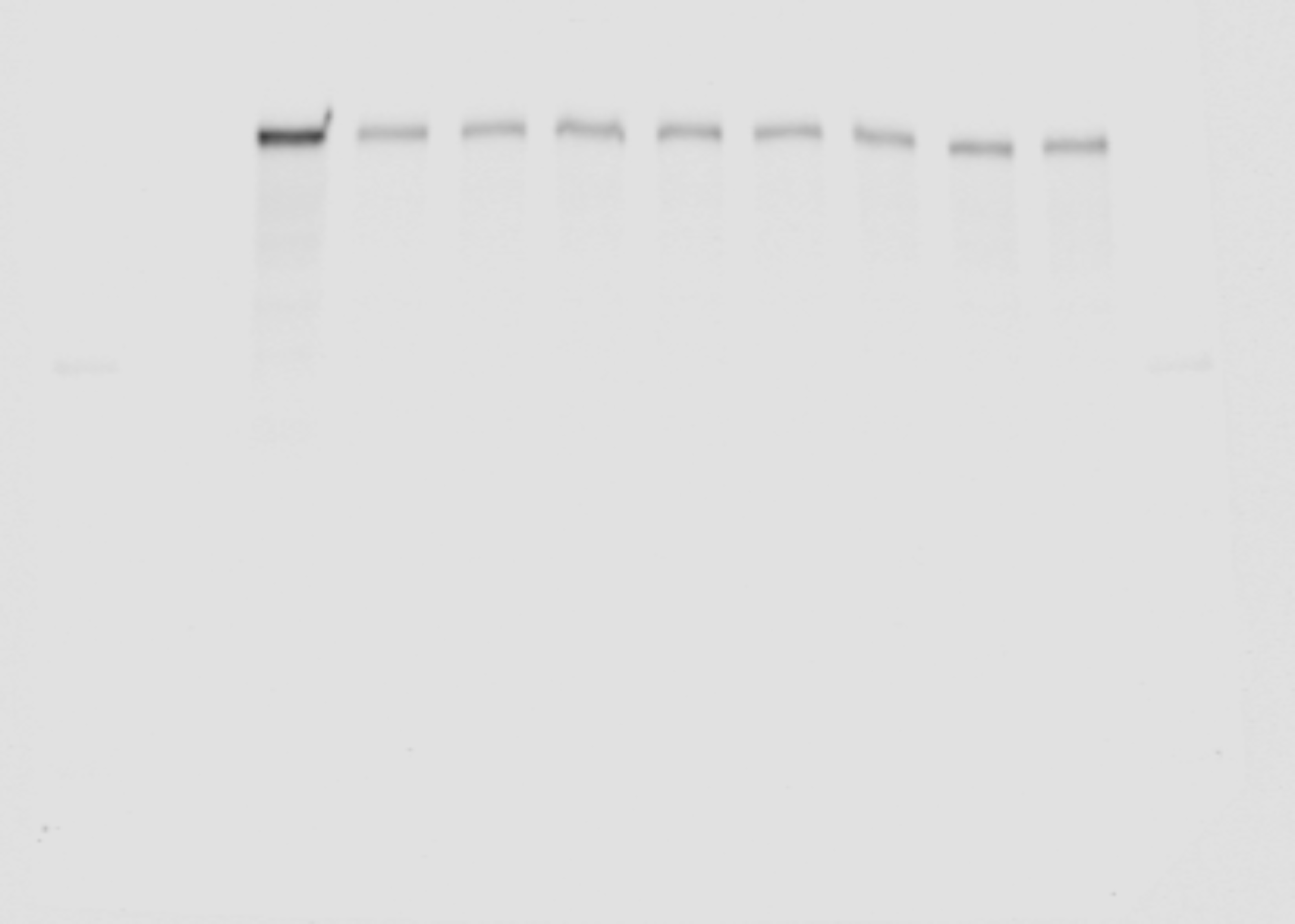

Supplement: Figure 6—figure supplement 1—source data 1. [file elife-77779-fig6-figsupp1-data1.zip › Figure 6-figure supplement 1 source data 1/Figure 6-figure supplement 1C-RawImages/NUP153.tif]

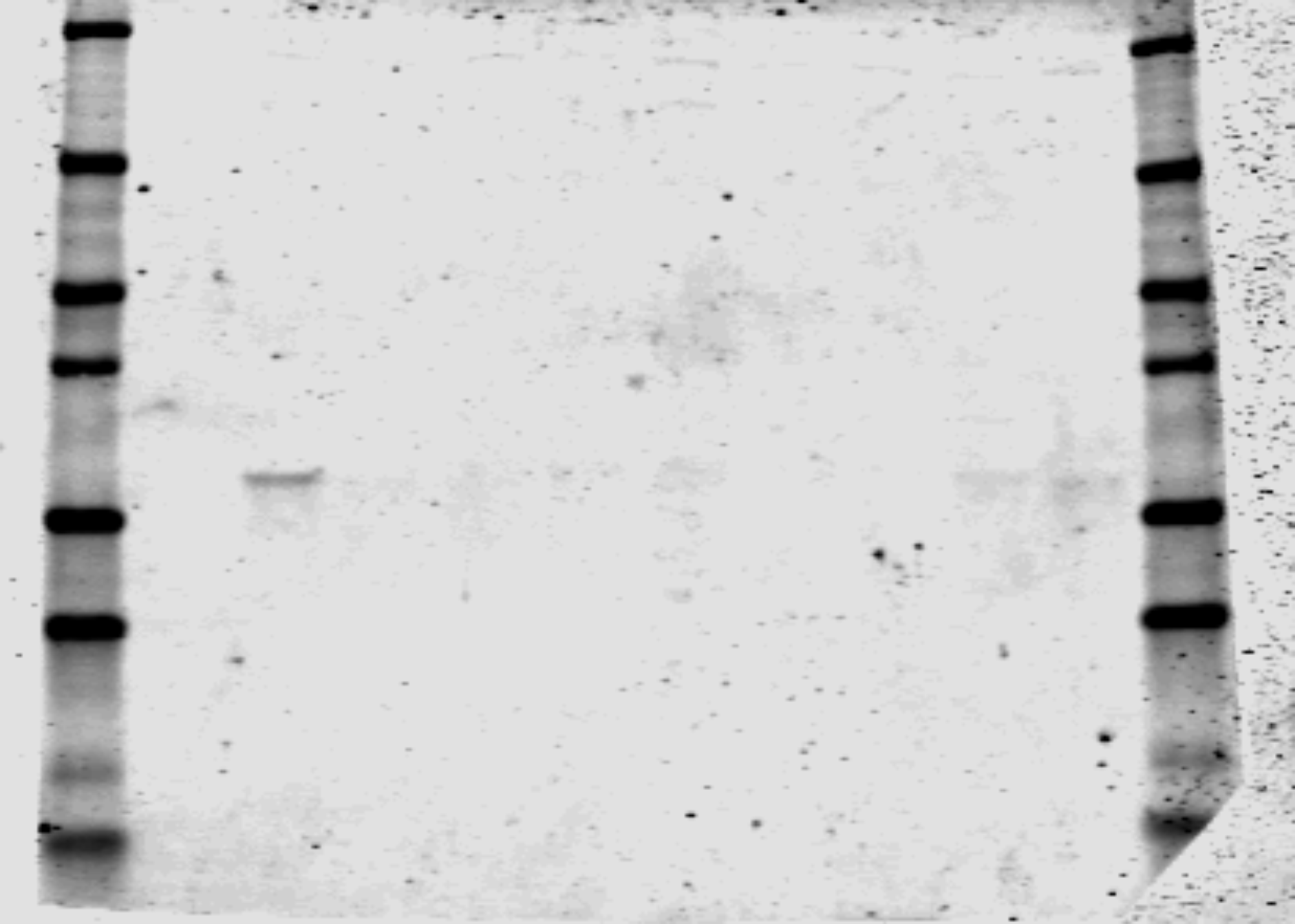

Supplement: Figure 6—figure supplement 1—source data 1. [file elife-77779-fig6-figsupp1-data1.zip › Figure 6-figure supplement 1 source data 1/Figure 6-figure supplement 1C-RawImages/NUP50.tif]

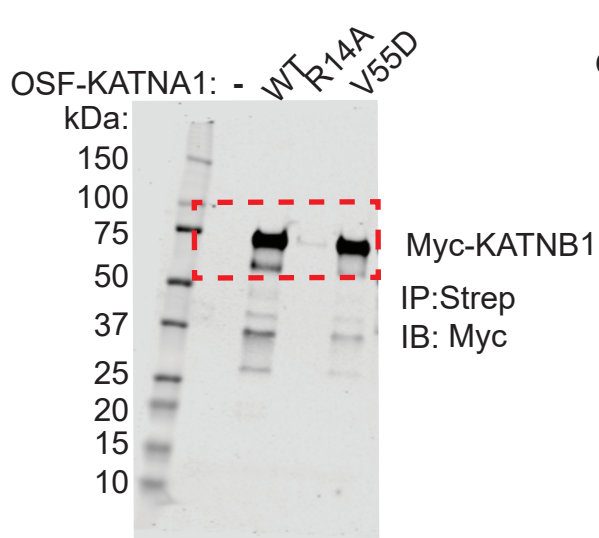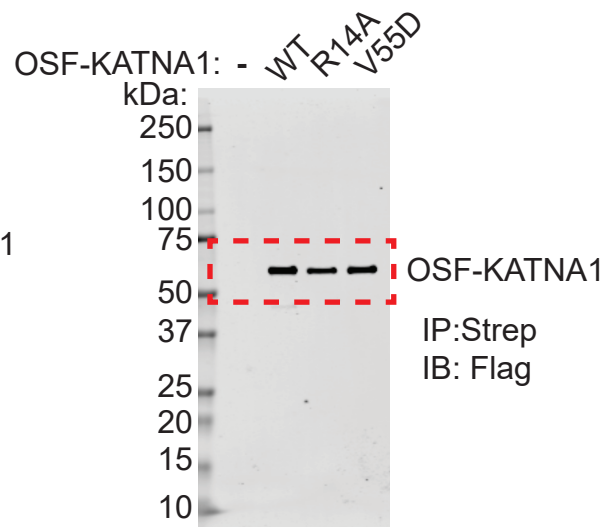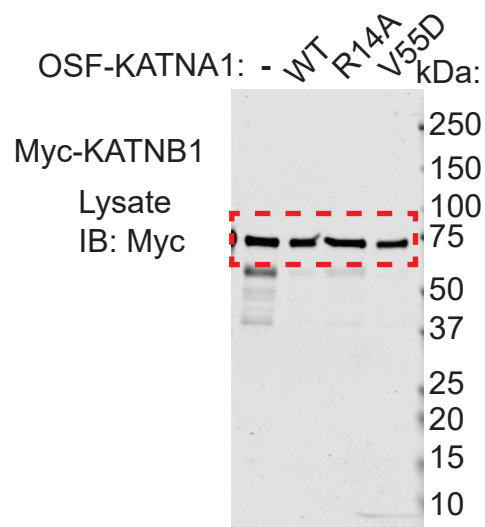

Uncropped Western blots for Figure 6-figure supplement 2B.

Supplement: Figure 6—figure supplement 2—source data 1. [file elife-77779-fig6-figsupp2-data1.zip › Figure 6-figure supplement2B source data 1/Figure6-figuresupplement-2B uncropped blots.pdf]

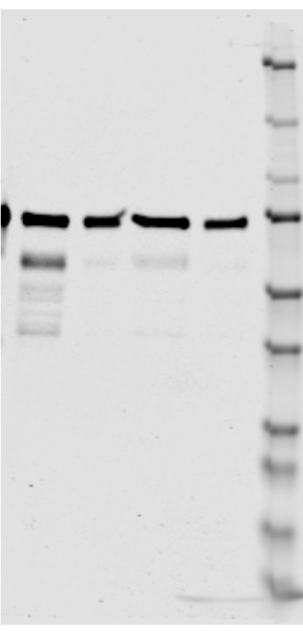

Supplement: Figure 6—figure supplement 2—source data 1. [file elife-77779-fig6-figsupp2-data1.zip › Figure 6-figure supplement2B source data 1/Raw images/Figure 6_figure supplement2BLysate_ProbeMyc.tif]

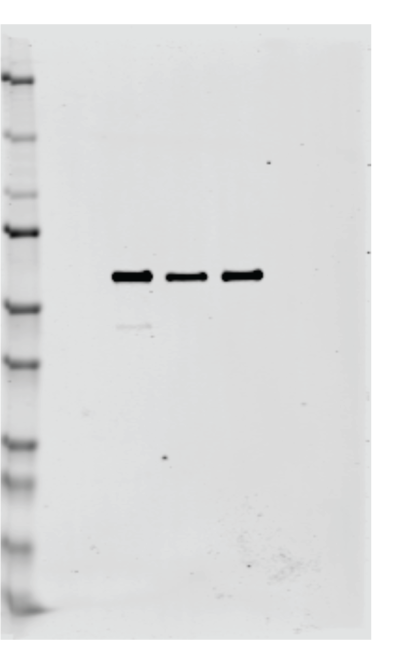

Supplement: Figure 6—figure supplement 2—source data 1. [file elife-77779-fig6-figsupp2-data1.zip › Figure 6-figure supplement2B source data 1/Raw images/Figure 6_figure supplementXBIPOSF_ProbeFlag.tif]

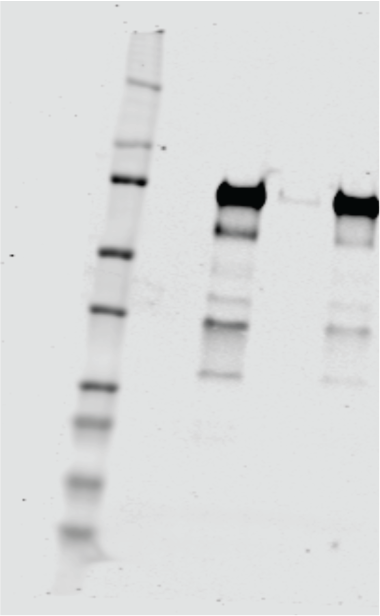

Supplement: Figure 6—figure supplement 2—source data 1. [file elife-77779-fig6-figsupp2-data1.zip › Figure 6-figure supplement2B source data 1/Raw images/Figure 6_figure supplement2BIPOSF_ProbeMyc.tif]
