# Supplementary material for: Comprehensive analysis of the human ESCRT-III-MIT domain interactome reveals new cofactors for cytokinetic abscission
Source: eLife. 2022 Sep 15;11:e77779. doi: 10.7554/eLife.77779 (PMC9477494; doi:10.7554/eLife.77779)
Supplement: Figure 5—figure supplement 1—source data 1. [file elife-77779-fig5-figsupp1-data1.zip › Figure 5-figure supplement 1-source data 1/Figure5_figuresupplement1B_uncroppedblots.pdf]

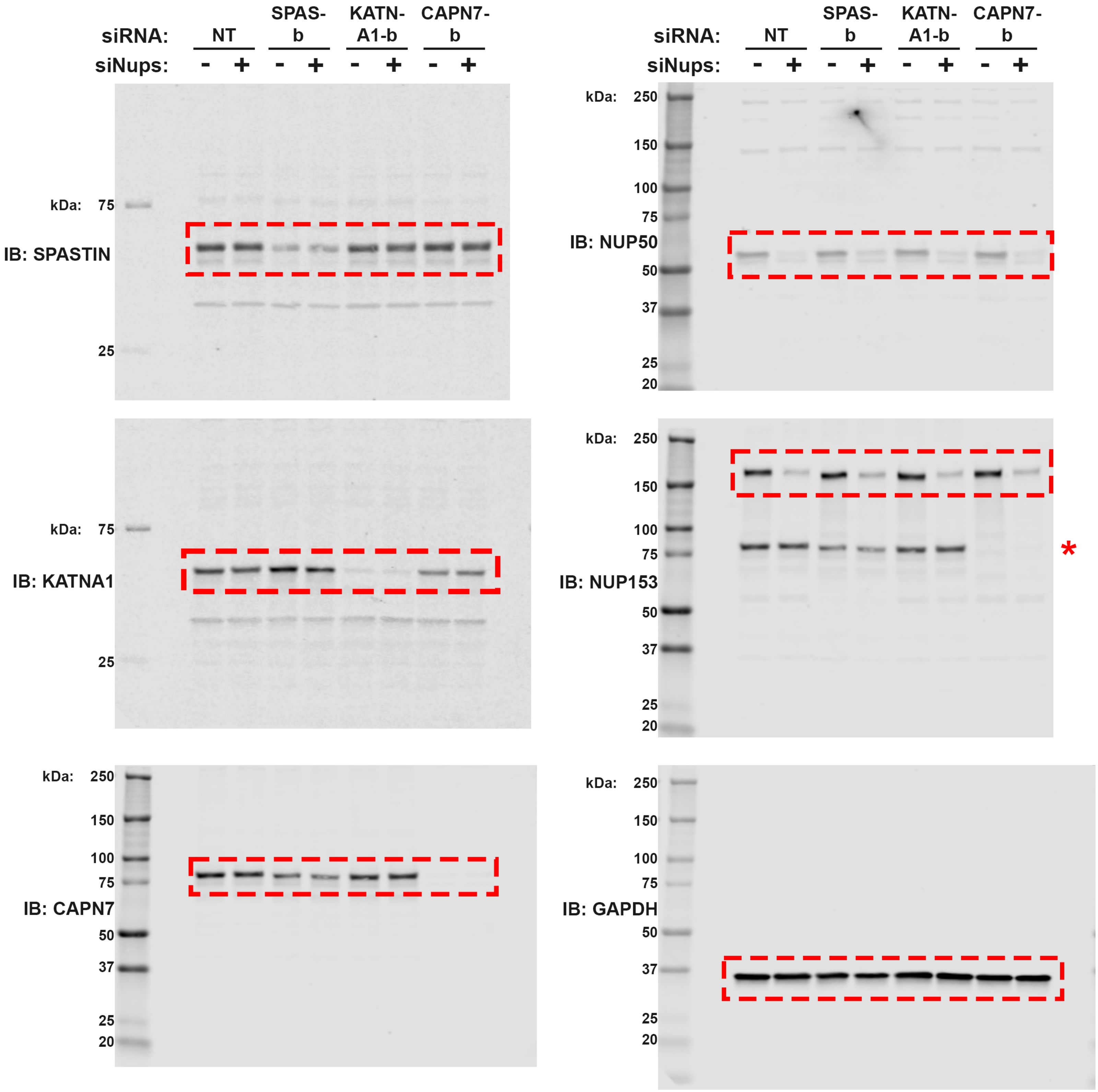

Uncropped Western blots for Figure 5-figure supplement 1B. The CAPN7 blot shown at the bottom left was reprobed with the NUP153 antibody (shown middle right). * indicates the residual CAPN7 signal.
