# Supplementary material for: Comprehensive analysis of the human ESCRT-III-MIT domain interactome reveals new cofactors for cytokinetic abscission
Source: eLife. 2022 Sep 15;11:e77779. doi: 10.7554/eLife.77779 (PMC9477494; doi:10.7554/eLife.77779)
Supplement: Figure 6—figure supplement 1—source data 1. [file elife-77779-fig6-figsupp1-data1.zip › Figure 6-figure supplement 1 source data 1/Figure 6-figure supplement 1A uncropped blots.pdf]

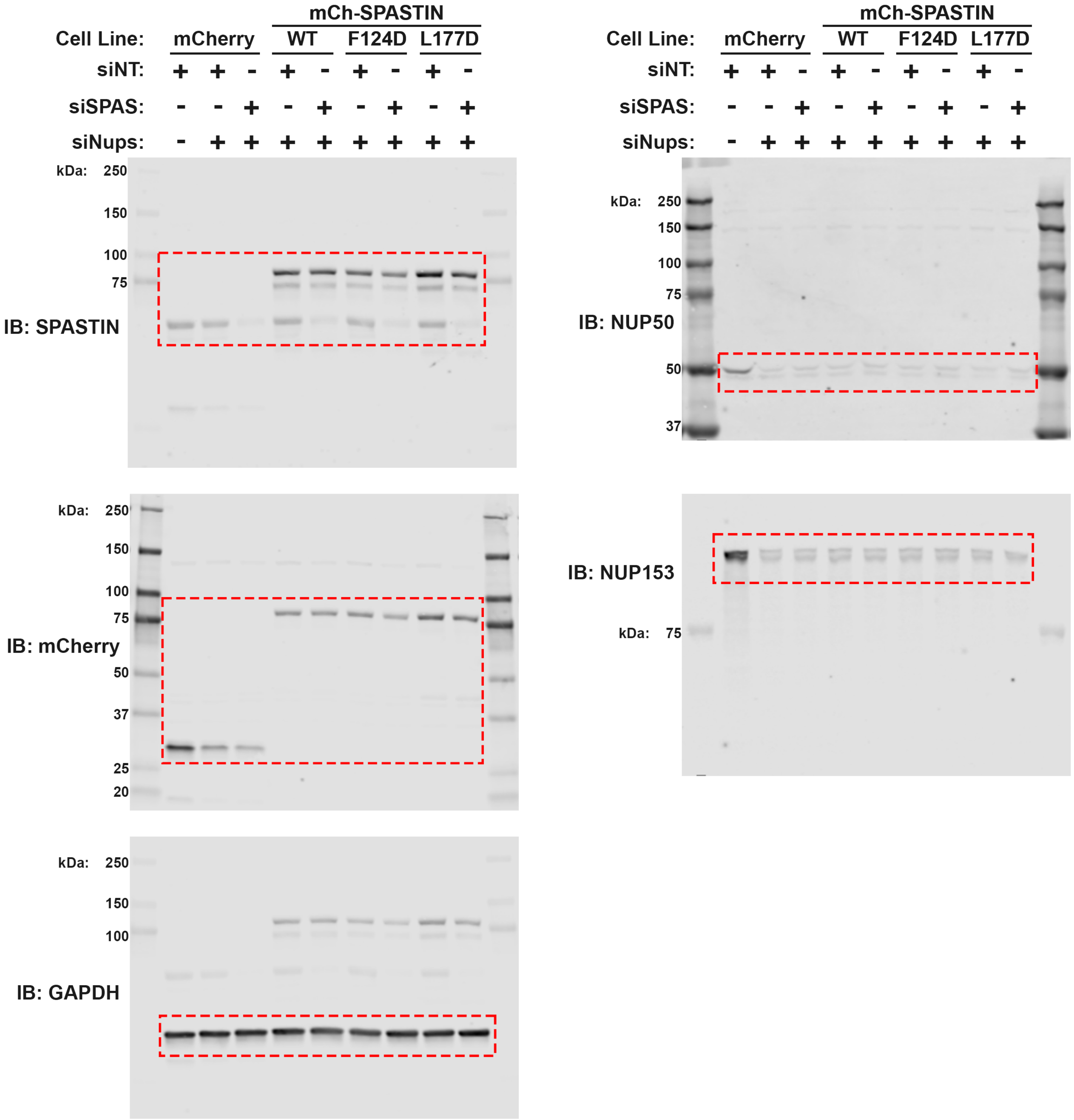

Uncropped Western blots for Figure 6 -figure supplement 1A.
