# Supplementary material for: Comprehensive analysis of the human ESCRT-III-MIT domain interactome reveals new cofactors for cytokinetic abscission
Source: eLife. 2022 Sep 15;11:e77779. doi: 10.7554/eLife.77779 (PMC9477494; doi:10.7554/eLife.77779)
Supplement: Supplementary file 1. — (A) MIT domain protein constructs used in our screen. (B) Fluorescently labeled ESCRT-III C-terminal tails used in the fluorescent polarization screen. (C) Unlabeled ESCRT-III C-terminal peptide tails used for competition experiments and structural biology. [file elife-77779-supp1.docx]

**Supplemental File 1A. MIT Domains**

| **MIT DOMAINS** | **Expected Mass (Da)** | **Measured Mass (Da)** | **Uniprot #** | **Notes** |
| --- | --- | --- | --- | --- |
| pCA528 AMSH (1-178) | 21172.0 | 21171.0 | O95630 |  |
| pCA528 AMSHLP (16-218) | 23973.2 | 23973.2 | Q96FJ0 |  |
| pGEX-THTAP CAPN7 (1-165) | 18871.0 | 18870.5 | Q9Y6W3 | GPH tag |
| pCA528 CAPN7 (1-165) | 18579.80 | 18581.34 | Q9Y6W3 |  |
| pCA528 CAPN7 V18D (1-165) | 18595.75 | 18595.35 | Q9Y6W3 |  |
| pCA528 CAPN7 F98D (1-165) | 18547.71 | 18547.35 | Q9Y6W3 |  |
| pCA528 KATNA1 (1-79) | 9429.9 | 9428.8 | A8K7S5 |  |
| pCA528 KATNA1-V55D (1-79) | 9445.8 | 9444.7 | A8K7S5 |  |
| pCA528 KATNAL1 (1-87) | 10098.5 | 10098.0 | Q9BW62 |  |
| pCA528 KATNAL2 (1-98) | 11681.35 | 11680.83 | Q8IYT4 |  |
| pGEX2T LIP5 (1-168) |  |  | Q9NP79 | Skalicky, 2012 |
| pGEX-THTAP MITD1 (1-85) | 9897.1 | 9897.1 | Q8WV92 | GPH tag |
| pGEX-THTAP NRBF2 (1-111) | 12785.0 | 12784.0 | Q96F24 | GPH tag |
| pCA528 RPS6KC1 (235-316) | 9313.9 | 9312.8 | Q96S38 |  |
| pCA528 RPS6KL1 (20-140) | 9512.8 | 9512.0 | Q9Y6S9 |  |
| pGEX-THTAP SNX15 (263-342) | 8675.9 | 8676.2 | Q9NRS6 | GP tag |
| pCA528 SNX15 (263-342) | 8521.5 | 8520.6 | Q9NRS6 |  |
| pGEX-THTAP SPARTIN (1-101) | 11697.0 | 11698.0 | Q8N0X7 | GPH tag |
| pCA528 SPASTIN WT (112-196) | 9763.2 | 9762.1 | Q9UBP0 |  |
| pCA528 SPASTIN WT (108-200) | 10687.3 | 10686.1 | Q9UBP0 |  |
| pCA528 SPASTIN F124D (108-200) | 10652.3 | 10653.0 | Q9UBP0 |  |
| pCA528 SPASTIN L177D (108-200) | 10689.3 | 10688.5 | Q9UBP0 |  |
| ULK3 (MIT)_2_ (277-449) |  |  | Q6PHR2 | Caballe, 2015 |
| pCA528 USP8 (1-147) | 17260.0 | 17260.0 | P40818 |  |
| pCA528 USP54 (731-878) | 16728.5 | 16727.4 | Q70EL1 |  |
| pET16b VPS4A (1-84) |  |  | Q9UN37 | Stuchell-Brereton,2007 |
| pGEX VPS4B (1-86) |  |  | O75351 | Stuchell-Brereton,2007 |
| pGEX-THTAP VPS9D1 (1-97) | 10657.2 | 10656.6 | Q9Y2B5 | GPH tag |

**Supplementary File 1B. Fluor-labeled ESCRT-III Peptides^1^**

| ESCRT-III | Sequence^1^ | Calculated Mass (Da) | Measured Mass (Da) |
| --- | --- | --- | --- |
| IST1  316-366 | **GC**PADNYDNFVLPELPSVPDTLPTASAGASTSASEDIDFDDLSRRFEELKKKT | 6196 | 6192 |
| IST1 C-Cys 316-366 | GPADNYDNFVLPELPSVPDTLPTASAGASTSASEDIDFDDLSRRFEELKKKT**GC** | 6271 | 6270 |
| IST1  MIM_316-343_ | **GC**PADNYDNFVLPELPSVPDTLPTASAGAS | 3480 | 3480 |
| IST1  MIM_344-366_ | **C**TSASEDIDFDDLSRRFEELKKKT | 3296 | 3296 |
| CHMP1A  140-196 | **GC**TTPQEQVDSLIMQIAEENGLEVLDQLSQLPEGASAVGESSVRSQEDQLSRRLAALRN | 6369 | 6365 |
| CHMP1B  143-199 | **GC**TTPQNQVDMLLQEMADEAGLDLNMELPQGQTGSVGTSVASAEQDELSQRLARLRDQV | 6347 | 6343 |
| CHMP2A  152-222 | **GC**GDEEDEEESDAVVSQVLDELGLSLTDELSNLPSTGGSLSVAAGGKKAEAAASALADADADLEERLKNLRRD | 7939 | 7939 |
| CHMP2B  141-213 | **GC**MINDTLDDIFDGSDDEEESQDIVNQVLDEIGIEISGKMAKAPSAARSLPSASTSKATISDEEIERQLKALGVD | 8448 | 8446 |
| CHMP3  159-222 | **GC**EEAEMEIDRILFEITAGALGKAPSKVTDALPEPEPPGAMAASEDEEEEEEALEAMQSRLATLRS | 7525 | 7525 |
| CHMP4A  153-222 | **GC**GDDVDEDELLEELEELEQEELAQELLNVGDKEEEPSVKLPSVPSTHLPAGPAPKVDEDEEALKQLAEWVS | 8375 | 8372 |
| CHMP4B  156-224 | **GC**GEEFDEDELMAELEELEQEELDKNLLEISGPETVPLPNVPSIALPSKPAKKKEEEDDDMKELENWAGSM | 8424 | 8423 |
| CHMP4C  156-233 | **GC**GDDFDEDELMAELEELEQEELNKKMTNIRLPNVPSSSLPAQPNRKPGMSSTARRSRAASSQRAEEEDDDIKQLAAWAT | 8907 | 8904 |
| CHMP5  148-219 | **GC**ALSRSYGTPELDEDDLEAELDALGDELLADEDSSYLDEAASAPAIPEGVPTDTKNKDGVLVDEFGLPQIPAS | 8187 | 8186 |
| CHMP6  145-201 | **GC**AGSFTQEDEDAILEELSAITQEQIELPEVPSEPLPEKIPENVPVKARPRQAELVAAS | 6835 | 6835 |
| CHMP7  366-453 | **GC**AGGVTNGLDFDSEELEKELDILLQDTTKEPLDLPDNPRNRHFTNSVPNPRISDAELEAELEKLSLSEGGLVPSSKSPKRQLEPTLKPL | 10315 | 10314 |

^1^Cysteines of non-native **GC** or **C** residues were used to couple Alexa Green 488 maleimide fluorescent dyes to the peptides.

**Supplemental File 1C. Unlabeled ESCRT-III Peptides for Crystallography and Competition Binding Assays**

| **ESCRT-III** | **Sequence** | **Calculated Mass (Da)** | **Measured Mass (Da)** |
| --- | --- | --- | --- |
| IST1  MIM_344-366_ | TSASEDIDFDDLSRRFEELKKKT | 2730.9 Da | 2730.4 Da |
| CHMP4B | GEEFDEDELMAELEELEQEELDKNLLEISGPETVPLPNVPSIALPSKPAKKKEEEDDDMKELENWAGSM | 7800.6 Da | 7799.7 Da |
| CHMP4C | GDDFDEDELMAELEELEQEELNKKMTNIRLPNVPSSSLPAQPNRKPGMSSTARRSRAASSQRAEEEDDDIKQLAAWAT | 8746.5 Da | 8746.2 Da |
| IST1  316-366 | GPADNYDNFVLPELPSVPDTLPTASAGASTSASEDIDFDDLSRRFEELKKKT | 5629.3 Da | 5628.7 Da |
