# Supplementary material for: Comprehensive analysis of the human ESCRT-III-MIT domain interactome reveals new cofactors for cytokinetic abscission
Source: eLife. 2022 Sep 15;11:e77779. doi: 10.7554/eLife.77779 (PMC9477494; doi:10.7554/eLife.77779)
Supplement: Supplementary file 2. — (A) Plasmids, (B), siRNA sequences, and (C) antibodies used for this study. [file elife-77779-supp2.docx]

**Supplemental File 2A. Plasmids**

| **Plasmid** | **Internal ID** | **DNASU# or Addgene #** | **Uniprot #** | **Source/**  **Reference** |
| --- | --- | --- | --- | --- |
| **MIT DOMAINS** |  |  |  |  |
| pCA528-AMSH (1-178) | WISP18-22 | 160050 (Addgene) | O95630 | This Study |
| pCA528 AMSHLP (16-218) | WISP20-83 | 180609 (Addgene) | Q96FJ0 | This Study |
| pGEX-THTAP- CAPN7 (1-165) | WISP21-129 | 180610 (Addgene) | Q9Y6W3 | This Study |
| pCA528 CAPN7 WT (1-165) | WISP21-25 | 180611 (Addgene) | Q9Y6W3 | This Study |
| pCA528 CAPN7 V18D (1-165) | WISP21-26 | 180612 (Addgene) | Q9Y6W3 | This Study |
| pCA528 CAPN7 F98D (1-165) | WISP21-27 | 180613 (Addgene) | Q9Y6W3 | This Study |
| pCA528 KATNA1 (1-79) | WISP19-20 | 180614 (Addgene) | A8K7S5 | This Study |
| pCA528 KATNA1-V55D (1-79) | WISP20-23 | 180615 (Addgene) | A8K7S5 | This Study |
| pCA528 KATNAL1 (1-87) | WISP20-54 | 180618 (Addgene) | Q9BW62 | This Study |
| pCA528 KATNAL2 (1-98) | WISP21-28 | 180619 (Addgene) | Q8IYT4 | This Study |
| pGEX2T-LIP5 (1-168) | WISP10-486 | HSCD00587773 | Q9NP79 | Skalicky, 2012 |
| pGEX-THTAP-MITD1 (1-85) | WISP20-35 | 180620 (Addgene) | Q8WV92 | This Study |
| pGEX-THTAP-NRBF2 (1-111) | WISP20-88 | 180621 (Addgene) | Q96F24 | This Study |
| pCA528 RPS6KC1 (235-316) | WISP20-87 | 180622 (Addgene) | Q96S38 | This Study |
| pCA528 RPS6KL1 (20-140) | WISP20-32 | 180623 (Addgene) | Q9Y6S9 | This Study |
| pGEX-THTAP-SNX15 (263-342) | WISP21-130 | 180624 (Addgene) | Q9NRS6 | This Study |
| pCA528-SNX15 (263-342) | WISP21-14 | 180625 (Addgene) | Q9NRS6 | This Study |
| pGEX-THTAP-SPARTIN (1-101) | WISP20-57 | 180626 (Addgene) | Q8N0X7 | This Study |
| pCA528 SPASTIN WT (112-196) | WISP21-11 | 180627 (Addgene) | Q9UBP0 | This Study; xray |
| pCA528 SPASTIN WT (108-200) | WISP21-10 | 180628 (Addgene) | Q9UBP0 | This Study; FP |
| pCA528 SPASTIN F124D (108-200) | WISP21-12 | 180629 (Addgene) | Q9UBP0 | This Study |
| pCA528 SPASTIN L177D (108-200) | WISP21-13 | 180634 (Addgene) | Q9UBP0 | This Study |
| pGEX-PP-ULK3 (MIT)_2_ (277-449) | WISP14-112 | HSCD00696230 | Q6PHR2 | Caballe, 2015 |
| pCA528 USP8 (1-147) | WSIP20-86 | 180636 (Addgene) | P40818 | This Study |
| pCA528 USP54 (731-878) | WISP20-85 | 180637 (Addgene) | Q70EL1 | This Study |
| pET16B VPS4A (1-84) | WISP05-43 | 180638 (Addgene) | Q9UN37 | Stuchell-Brereton, 2007 |
| pGEXET16B VPS4B (1-86) | WISP04-155 | 180639 (Addgene) | O75351 | Stuchell-Brereton, 2007 |
| pGEX-THTAP VPS9D1 (1-97) | WISP20-89 | 180640 (Addgene) | Q9Y2B5 | This Study |
|  |  |  |  |  |
| **ESCRT-III PEPTIDES** |  |  |  |  |
| pCA528 CHMP1A (140-196) | WISP18-1 | 104593 (Addgene) | Q9HD42 | This Study |
| pCA528 CHMP1B (143-199) | WISP18-2 | 108255 (Addgene) | Q7LBR1 | This Study |
| pCA528 CHMP2A (152-222) | WISP18-3 | 104594 (Addgene) | O43633 | This Study |
| pCA528 CHMP2B (141-213) | WISP18-4 | 104595 (Addgene) | Q9UQN3 | This Study |
| pCA528 CHMP3 (159-222) | WISP18-5 | 104599 (Addgene) | Q9Y3E7 | This Study |
| pCA528 CHMP4A (153-222) | WISP18-6 | 108256 (Addgene) | Q9BY43 | This Study |
| pCA528 CHMP4B (156-224) | WISP18-7 | 104596 (Addgene) | Q9H444 | This Study |
| PCA528 CHMP4B (156-224; no Cys) | WISP20-140 | 180641 (Addgene) | Q9H444 | This Study |
| pCA528 CHMP4C (156-233) | WISP18-9 | 108257 (Addgene) | Q96CF2 | This Study |
| pCA528 CHMP4C (156-233; no Cys) | WISP20-14 | 180642 (Addgene) | Q96CF2 | This Study |
| pCA528 CHMP5 (148-219) | WISP18-10 | 108259 (Addgene) | Q9NZZ3 | This Study |
| pCA528 CHMP6 (145-201) | WISP18-11 | 104597 (Addgene) | Q96FZ7 | This Study |
| pCA528 CHMP7 (366-453) | WISP18-12 | 104598 (Addgene) | Q8WVX9 | This Study |
| pCA528 IST1 (316-366) | WISP14-121 | HSCD00696258 | P53390 | Caballe, 2015 |
| pCA528 IST1 (316-366; no Cys) | WISP20-99 | 180643 (Addgene) | P53390 | This Study |
| pCA528 IST1 (316-366) C-Cys | WISP20-100 | 191234(Addgene) | P53390 | This Study |
|  |  |  |  |  |
| **Mammalian Expression Vectors** |  |  |  |  |
| pCAG-OSF-PP-ULK1(MIT)_2_ (833-1050) | WISP20-37 | 180644 (Addgene) | O75385 | This Study |
| pCAG-OSF-ULK3(MIT)_2_ (277-449) | WISP14-112 | HSCD00696230 | Q6PHR2 | Caballe, 2015 |
| pCAG-CHMP1A-myc | WISP08-77 | HSCD00520978 | Q9HD42 | Caballe, 2015 |
| pCAG-CHMP1B-myc | WISP08-78 | HSCD00696225 | Q7LBR1 | Caballe, 2015 |
| pCAG-CHMP2A-myc | WISP08-97 | HSCD00696226 | O43633 | Caballe, 2015 |
| pCAG-CHMP2A-myc L216D/L219D | WISP20-13 | 180645 (Addgene) | O43633 | This Study |
| pCAG-Myc-IST1 | WISP07-77 | HSCD00751713 | P53390 | Caballe, 2015 |
| pCAG-OSF empty | WISP06-65 |  |  |  |
| pRK5-Myc-ATG13 | WISP20-30 | 31965 (Addgene) | O75143 | Jung, 2009 |
| pCAG-Myc-KATNB1 | WISP20-16 | 160054 (Addgene) | Q9BVA0 | This Study |
| pCAG-OSF-KATNA1 WT | WISP20-18 | 160051 (Addgene) | A8K7S5 | This Study |
| pCAG-OSF-KATNA1 V55D | WISP20-17 | 160053 (Addgene) | A8K7S5 | This Study |
| pCAG-OSF-KATNA1 R14A | WISP20-19 | 160052 (Addgene) | A8K7S5 | This Study |
| pLVX-TetOn-Advanced |  |  |  | Clontech/ Don Ayer gift |
| pLVX-tight-puro |  |  |  | Clontech/  Don Ayer gift |
| pLVX-mCherry | WISP20-61 | 180646 (Addgene) |  | This Study |
| pLVX-mCherry-SPASTIN | WISP20-43 | 180647 (Addgene) | Q9UBP0 | This Study |
| pLVX-mCherry-SPASTIN-F124D | WISP20-44 | 180648 (Addgene) | Q9UBP0 | This Study |
| pLVX-mCherry-SPASTIN-L177D | WISP20-45 | 180649 (Addgene) | Q9UBP0 | This Study |
| pLVX-mCherry-KATNA1 | WISP20-47 | 180650 (Addgene) | Q9BVA0 | This Study |
| pLVX-mCherry-KATNA1-V55D | WISP20-48 | 180651 (Addgene) | Q9BVA0 | This Study |
| pLVX-CAPN7-mCherry | WISP20-50 | 180652 (Addgene) | Q9Y6W3 | This Study |
| pLVX-CAPN7-F98D-mCherry | WISP20-53 | 180653 (Addgene) | Q9Y6W3 | This Study |

**Supplemental File 2B. siRNA Sequences**

| **siRNA** | **Protein Target** | **Sense Sequence** | **Source** | **Reference** |
| --- | --- | --- | --- | --- |
| siCHMP4C (siC4C) | CHMP4C | CACUCAGAUUGAUGGCACA | U of U Cores | Strohacker, 2021 |
| siCAPN7-2213 (siCAPN7) | CALPAIN7 | GCACCCAUACCUUUACAUU | U of U Cores | This Study |
| siCAPN7-2444 (siCAPN7-b) | CALPAIN7 | GGCCGUUACUGAUUGAGCU | Thermo Fisher | Cat # s23893 |
| siIST1-490 | IST1 | AGAUACCUGAUUGAAAUUG | U of U Cores | Bajorek, 2009 |
| siKATNA1-583 (siKATNA1) | KATNA1 | GGACAGCACUCCCUUGAAA | U of U Cores | This Study |
| siKATNA1-pool (siKATNA1-b) | KATNA1 | ON-TARGET-Plus siRNA SMARTPool | Horizon Discovery | Cat # L-005157-02-005 |
| siNT | Non-targeting | GCAAAUCUCCGAUCGUAGA | U of U Cores | Mackay, 2010 |
| siNUP50 | Nup50 | GGAGGACGCUUUUCUGGAU | U of U Cores | Mackay, 2010 |
| siNUP153 | Nup153 | GGACUUGUUAGAUCUAGUU | U of U Cores | Mackay, 2010 |
| siSPAS-726 (siSPAS) | SPASTIN | GAACAGUGUGAAAGAGCUA | U of U Cores | This study |
| siSPAS-4146 (siSPAS-b) | SPASTIN | CGUUAUUGAUACUUGGAUA | U of U Cores | This study |

**Supplemental File 2C. Antibodies**

| **Target** | **Host Organism** | **Source** | **Product Number** | **Application^1^** | **Dilution Factor** |
| --- | --- | --- | --- | --- | --- |
| CAPN-7 | Rabbit | Proteintech | 26985-1-AP | IF  WB | 1:1000  1:5000 |
| CEP55 | Sheep | Bastos and Barr, 2010 | N/A | IF | 1:3500 |
| CHMP4C | Rabbit | Sadler, M.-Serrano, 2018 | N/A | WB | 1:500 |
| GAPDH | Mouse | Millipore | MAB374 | WB | 1:20,000 |
| IST1 | Rabbit | Sundquist Lab/ Covance | UT560 | WB | 1:1000 |
| KATNA1 | Rabbit | Proteintech | 17560-1-AP | IF | 1:1000 |
| KATNA1 | Rabbit | Abcam | ab111881 | IF  WB | 1:500  1:1000 |
| mCherry | Rabbit | Abcam | ab167453 | IF | 1:2000 |
| mCherry | Rabbit | Novus Biologicals | NBP2-25157 | WB | 1:1000 |
| NUP153 (SA1) | Mouse | Brian Burke, Singapore | N/A | WB | 1:50 |
| NUP50 | Rabbit | Mackay, et al., 2010 | N/A | WB | 1:2500 |
| SPASTIN | Mouse | Sigma | S7074 | IF  WB | 1:1000  1:1000 |
| α-TUBULIN (DM1A) | Mouse | Cell Signaling Technology | 3873S | IF | 1:2500 |
| Myc | Mouse | EMD Millipore | Clone 4A6 | WB | 1:2500 |
| Flag | Mouse | Sigma | M2 | WB | 1:10,000 |
| IRDye 800 CW,  Mouse | Donkey | Licor | 926-32212 | WB | 1:10,000 |
| IRDye 680,  Mouse | Donkey | Licor | 926-68072 | WB | 1:10,000 |
| IRDye 800 CW,  Rabbit | Donkey | LiCor | 926-32213 | WB | 1:10,000 |
| IRDye 680, Rabbit | Donkey | LiCor | 926-68073 | WB | 1:10,000 |
| Alexa Fluor Plus 647, Mouse IgG | Donkey | Thermo Fisher | A-32787 | IF | 1:1000 |
| Alexa Fluor Plus 405, Rabbit IgG | Donkey | Thermo Fisher | A-48258 | IF | 1:1000 |
| Alexa Fluor Plus 594, Rabbit IgG | Donkey | Thermo Fisher | A-32754 | IF | 1:1000 |
| Alexa Fluor Plus 647, Rabbit IgG | Donkey | Thermo Fisher | A-32795 | IF | 1:1000 |
| Alexa Fluor 488,  Sheep IgG | Donkey | Thermo Fisher | A-11015 | IF | 1:1000 |

^1^IF = Immunofluorescence; WB = Western Blot
