# Supplementary material for: Comprehensive analysis of the human ESCRT-III-MIT domain interactome reveals new cofactors for cytokinetic abscission
Source: eLife. 2022 Sep 15;11:e77779. doi: 10.7554/eLife.77779 (PMC9477494; doi:10.7554/eLife.77779)
Supplement: Supplementary file 3. [file elife-77779-supp3.docx]

## Supplementary File 3. SPASTIN MIT-IST1 Complex Data Collection and Refinement Statistics

| **Data** | PDB 7S7J |
| --- | --- |
| **Crystal** | SPASTIN MIT (112-195):IST1 (344-366) |
| **Source, Wavelength (**Å**), Collection Date** | SSRL 9-1, 0.97946, 03-13-2016 |
| **Space group, unit cell constants (**a b c α β γ) | I4_1_, (79.614 79.614 36.331 90 90 90) |
| Resolution range | 28.15-1.15 (1.191-1.15) |
| Total reflections | 519,763 |
| Unique reflections | 38,262 (3250) |
| Multiplicity | 13.6 (12.3) |
| Completeness (%) | 94.19 (80.39) |
| Mean I/sigma (I) | 16.4 (4.0) |
| Wilson B-factor | 10.40 |
| R-merge | 0.103 (1.647) |
| R-meas | 0.107 (1.718) |
| R-pim | 0.029 (0.480) |
| CC1/2 | 0.988 (0.865) |
|  |  |
| Refinement |  |
| Program | Phenix.refine, version 1.17.1 |
| Reflections used in refinement | 38,243 (3239) |
| Reflections used for R-free | 2001 (170) |
| R-work | 0.1494 (0.1969) |
| R-free | 0.1586 (0.1886) |
| Number of non-hydrogen atoms | 998 |
| macromolecules | 861 |
| ligands | 25 |
| solvent | 112 |
| Protein residues | 102 |
| RMS (bonds), (angles) | 0.007, 0.90 |
| Ramachandran favored, allowed, outliers (%) | 100.00, 0.00, 0.00 |
| Rotamer outliers (%) | 1.10 |
| Clashscore | 2.22 |
| Average B-factor (Å^2^) | 22.12 |
| macromolecules | 20.60 |
| ligands | 28.80 |
| solvent | 32.36 |
| Number of TLS groups | 1 |

Statistics for the highest-resolution shell are shown in parentheses.
